# Cannabis concentrate vaping chemistry

**DOI:** 10.3389/ftox.2025.1568207

**Published:** 2025-06-09

**Authors:** Kaelas R. Munger, Killian M. Anreise, Robert M. Strongin

**Affiliations:** Department of Chemistry, Portland State University, Portland, OR, United States

**Keywords:** vaping, dabbing, cannabis concentrates, terpenes, ketene, pine rosin, cannabis vaping, terpene vaping

## Abstract

**Background:**

This review article addresses the vaping chemistry of manufactured cannabis concentrates—a topic that remains under-researched despite the widespread availability and growing popularity of these products. Given their current prevalence and the fact that many of the findings discussed herein are from early-stage investigations, further research is essential to fully assess the public health risks associated with concentrate use. The purpose of this article is to help begin to bridge this knowledge gap by outlining the technical challenges of studying cannabis concentrates and to present evidence-based data concerning toxicant exposures as a foundation for future investigations.

**Methods:**

A search of cannabis concentrate vaping within the date range of 2019-2025 on Google Scholar returned approximately 2,700 hits. A cannabis concentrate was defined as a sample containing at least 50% (w/w) cannabinoids. In addition to our group’s articles, the search results contained six manuscripts that described at least a partial focus on molecular emissions specifically derived from vaping or dabbing samples that included cannabis concentrates.

**Findings:**

Studying cannabis concentrate vaping poses distinct technical challenges that differ from those associated with electronic nicotine delivery systems. Emissions from vaping concentrates contain a substantial proportion of harmful aerosol toxicants, including isoprene, 3-methylcrotonaldehyde, 3-methyl-1-butene, and 2-methyl-2-butene. Moreover, some concentrate formulations have contained hazardous additives such as pine rosin and ketene precursors such as cannabinoid acetates. As with nicotine vaping, the presence of oxygen plays a critical role in driving the formation of many toxic chemical degradation products during vaping.

**Conclusion:**

Since the legalization of recreational cannabis, concentrates have become one of the most rapidly expanding segments of the U.S. cannabis market. However, research into the specific health risks of vaping these products has significantly lagged their widespread use. The studies presented in this review article highlight the potential for exposure to known toxicants during the vaping of cannabis concentrates.

## Introduction

Despite the current prevalence of cannabis vaping, substantive knowledge of its associated acute and chronic health effects is lacking. Although it is well-known that cannabis can harm the developing brain and other organs, (Batalla et al., 2013)^,^ (Sciences et al., 2017) its usage is relatively common among adolescents in the U.S. (Miech et al., 2023) In addition, vaping is emerging as an increasingly popular cannabis delivery mode with young people. For example, the prevalence of past 30-day cannabis vaping among 12th graders in the U.S. increased from 4.9% in 2017 to 14.8% in 2022. (Miech et al., 2023). Market projections indicate that the use of cannabis concentrates is rising at a significantly faster rate than that of flower or edibles. However, there is a notable lack of longitudinal studies on concentrate use, as well as a shortage of up-to-date prevalence and demographic data concerning the expanding population of concentrate users. (Bidwell et al., 2021).

Cannabis vaping encompasses the aerosolization of multiple product types, including dry plant material, cannabis concentrates (CCs), and cannabis-infused liquids or oils. Vaping liquids are formulated either by extracting plant constituents into e-cigarette solvents such as propylene glycol (PG) or glycerol (GL), or by dissolving CCs into these carriers. Alternatively, undiluted CCs—characterized by their viscous, oil-like nature—may be vaporized without the use of solvents. Concentrates are typically classified according to their physical properties or method of production; for instance, “rosin” refers to a solventless extract obtained through mechanical compression (heat and pressure), whereas butane hash oil (BHO) is produced via hydrocarbon (butane) extraction techniques. Depending on the manufacturing process, CCs may preserve the full spectrum of naturally occurring cannabinoids and terpenes or consist primarily of purified Δ^9^-tetrahydrocannabinol (Δ^9^-THC) reconstituted with commercial terpene blends and other excipients (e.g., distillates or isolates) (Morgan et al., 2022). Notably, while elevated systemic exposure to Δ^9^-THC has been associated with an increased risk of acute intoxication and psychosis (Di Forti et al., 2019), ∆^9^-THC levels in commercial CCs are typically >50-95% (Bidwell et al., 2021). This is a significantly higher concentration than the ∼10-30% ∆^9^-THC content found in plant material (ElSohly et al., 2016; Leung et al., 2021).

Proponents of vaping CCs claim that the aerosols produced are less harmful than the smoke from burning cannabis flower. This belief is based on the absence of combustion during vaping (Gieringer, 2001). However, the lack of evidence-based data concerning the potential risks of vaping concentrate aerosols is troublesome. This is particularly concerning in the case of vulnerable cohorts such as teens, pre-teens, chronically ill and elderly patients with compromised immune systems. A recent report includes evidence that CC vaping is associated with relatively greater risk of severe cannabis use disorder outcomes compared to more traditional routes of administration (Inman and Cservenka, 2024).

CC vaping has been less studied compared to other forms of cannabis vaping and smoking. To begin to understand the potential risks of vaping CCs while longer-term epidemiological studies are underway, it is necessary to investigate health-related chemical and physical properties of CCs. This includes understanding the toxic chemical emissions arising from CC heating and aerosolization.

## Methods

This review primarily highlights our group’s ongoing research into the chemistry of vaping and dabbing cannabinoids, terpenes, in the context of cannabis concentrates (CCs). A Google Scholar search of “cannabis concentrate vaping” within the 2019–2025 timeframe returned approximately 2,700 results. The search was further refined by examination of the citations within the references shown in Supplementary Table S1, as well as from their forward citations. While a number of publications were found that included aspects of CC vaping chemistry, most did not focus on this topic as a primary research aim. Instead, relevant information was often embedded within broader studies. Accordingly, selected findings from these studies have been integrated in Supplementary Table S1 and/or in the text where pertinent to support and contextualize the main theme of the review. This underscores a significant knowledge gap regarding the chemistry and toxicant exposures associated with vaping concentrates.

For instance, Supplementary Table S1 includes just six studies from other research groups that examine specific aspects of CC aerosol chemistry. The first entry reports the total amount of volatile organic compounds (VOCs), and on identifying carbon monoxide (CO), and carbon dioxide (CO_2_) in cannabis aerosols (Marrocco et al., 2022). The second describes the identification of aerosol components generated from concentrated THC oil and the chemical pathways leading to their formation. This study does include relevant radical oxidation and direct thermal decomposition chemical pathways. However, the main focus is on vitamin E acetate (VEA) vaping, and the precise contents of the commercial THC oil used are not clear (Li et al., 2022). The third study investigates cannabidiol quinone (CBDQ) and other select cannabinoids in both vaped and non-vaped commercial CBD distillate oils (Love et al., 2023). The fourth examines the formation of reactive oxygen species (ROS) from vaping medium-chain triglycerides (MCT), vitamin E acetate (VEA), and cannabis-containing cartridges using *in vitro* models (Muthumalage et al., 2020). The fifth is focused on analytical method development and describes the analysis of four carbonyls in aerosols from a variety of e-cigarette or vaping use-associated lung injury (EVALI) patient samples of widely varying compositions (McGuigan et al., 2022). The sixth also features an analysis of patients’ and other commercial samples, and determined additives, cannabinoids and terpenes in the liquids. Aerosol analysis also led to identifying terpenes and minor cannabinoids produced during aerosolization. (Guo et al., 2021).

Supplementary Table S1 also includes a second group of studies that analyzed aerosols from vaping highly diluted concentrates. The products studied—typically containing around 10% (w/w) cannabinoids—are markedly different from CCs. They feature significantly lower potency and include diluents such as propylene glycol (PG) and glycerol (GL) that are not characteristic of CC formulations. Entries 10-26 in Supplementary Table S1 describe molecular profiling of unvaped CCs to determine their contents. The remaining Table entries show recent citations on VEA and diluent vaping, indoor vaping, storage and decomposition chemistry, as well device properties. Not included herein are investigations on toxic metals in related cannabis products.

Overall, the results of the literature search demonstrate that studies focused on understanding the chemistry and specific chemical origins of the toxicant emissions from CC vaping are relatively sparse. To help address this literature gap, this review focuses on studies that systematically analyze the vaping chemistry of cannabinoids and terpenes, as well as their mixtures as CCs, and their impact on toxicant formation and emissions. Understanding the precise origin of toxicant exposures is an important step in harm mitigation.

### The emergence of cannabis vaping

Aerosolization to consume cannabis flower, as opposed to CCs, was first described in 2001 (Gieringer, 2001), predating the rise of tobacco e-cigarettes. Initial studies included handheld or tabletop devices that generated hot air blown over ground cannabis plant material to produce an aerosol for inhalation (Gieringer, 2001; Hazekamp et al., 2006) Gieringer et al. were the first to characterize the aerosol components emitted by a cannabis flower vaporizer, the Volcano^®^ tabletop vaporizer (Gieringer, 2001). They reported that vaping reduced the formation of harmful chemical degradation products compared to smoking. Follow-up research included various *in vitro* studies (Lanz et al., 2016), as well as small pre-clinical trials with human cohorts (Abrams et al., 2007; Earleywine and Barnwell, 2007; Wilsey et al., 2013).

The first report of an e-cigarette used to aerosolize cannabis appeared in 2011 (Etter and Bullen, 2011), with an internet survey and a literature review on the topic following in 2015 (Etter, 2015; Giroud et al., 2015). These reports revealed that cannabis vaping at the time largely involved do-it-yourself (DIY) approaches, including hazardous butane extraction methods, to produce CC material (Etter, 2015; Giroud et al., 2015).

Vape pens are a popular class of CC vaporizers. They are relatively small, possessing a battery and a cartridge. A pre-filled cartridge serves as a reservoir that contains the CC and a heating element. Dabbing is an alternative type of vaping that is uniquely associated with cannabis (Mullins, 2021). It involves the flash vaporization of a CC on a hot surface.

The precise origins of dabbing as a cannabis consumption method are unclear, but its mention in the literature appeared in a 2014 internet survey assessing user perceptions (Loflin and Earleywine, 2014). The survey data showed that dabbing led to elevated ∆^9^-THC tolerance, and was perceived as riskier than other consumption methods. (Loflin and Earleywine, 2014) Additional relatively early studies focused on cannabinoid aerosol transfer and tetrahydrocannabinolic acid (THCA) decarboxylation efficiency during dabbing. One involved the evaluation of the transfer efficiency of cannabinoids during dabbing (Elzinga et al., 2015). It was determined that ∼50% of the available ∆^9^-THC was transferred, depending on the type of cannabis extract used, and that THCA decarboxylation proceeded with over 90% conversion. Although initial surveys indicated some user hesitation about dabbing (Loflin and Earleywine, 2014), it has rapidly become a popular method for consuming cannabis concentrates (Mullins, 2021). Data from 2022 showed that 22.23% of adult and 23.39% of adolescent cannabis users reported past year CC dabbing (Leal and Moscrop-Blake, 2024).

### Technical challenges

An initial investigation of the release of toxic chemical degradation products from vaping a cannabis extract was published in 2016 (Varlet et al., 2016). The authors produced butane hash oil (BHO), mixed it with the tobacco e-cigarette solvent propylene glycol (PG), and aerosolized the mixture using a tobacco e-cigarette. They detected no volatile organic compounds (VOCs) other than two carbonyls, formaldehyde and acetaldehyde, while noting difficulties in dissolving BHO in PG, having achieved BHO solutions of only up to 10%. It is thus not clear if the aldehyde toxicants were formed from the heating and aerosolization of PG, which is well-known to produce toxic aldehydes upon vaping, or from the BHO. The authors noted the practicality of vaping cannabis with an e-cigarette for microdosing purposes (Varlet et al., 2016). Recently, other reports of ∼10% (w/w) cannabinoid solutions have surfaced (Sambiagio et al., 2023; Kim et al., 2025) in addition to a study of the aerosol compositions from vaping several cannabinoids at 50 mg/mL (∼5% w/v) concentrations in a fourth generation e-cigarette (Strongin, 2019) with PG/GL solvent (Robertson et al., 2024).

To begin investigating CC vaping without any solvent and dilution, we determined that a 9:1 THC:terpene ratio afforded practical samples for study in cannabis vaporizers. Terpenes are the second most abundant class of compounds in CCs, and, apart from adding sensorial as well as purported enhanced psychoactive (e.g., “entourage”) effects (Schlag et al., 2021), they moderate cannabinoid viscosity. However, we have found that too high a terpene ratio affords a material that is too fluid and leaks out of a vape pen (Meehan-Atrash et al., 2019). The 9:1 THC:terpene ratio afforded us a useful CC model material that was neither too viscous nor too fluid.

Although the 9:1 THC:terpene ratio is within the range of commercial CC formulations, for research purposes we also need to study the aerosolization of control compounds, such as pure cannabinoids or pure terpenes, to deconvolute the contribution of each CC component to the production of specific aerosol toxicants. To address this issue, we turned to dabbing because it allows aerosolization to take place on an exposed surface without reliance on wicking (Meehan-Atrash et al., 2019). This enabled heating and aerosolization of pure cannabinoids and pure terpenes to be investigated without confounding viscosity effects. In the tobacco e-cigarette vaping field, it is well-known that sample viscosity can have a deleterious effect on wicking efficiency (Strongin, 2019). Inefficient wicking is a major cause of toxicant formation during vaping since it can result in exposure of a sample to excessive temperatures via contact with a dry region of a heating element. Cannabinoid viscosity is not only a challenge for researchers but also has promoted the use of potentially harmful additives by manufacturers. For example, the use of a viscosity modifier (vitamin E acetate, VEA) as a CC additive has been associated with the e-cigarette or vaping use-associated lung injury (EVALI) outbreak in 2019, *vide infra* (Blount et al., 2020). The use of the dabbing technique for cannabis research informs the potential hazards of dabbing. However, it also enables one to better decouple the production of aerosol toxicants due to viscosity and/or aberrant wicking effects from toxicant production due to the chemical reactivity of cannabinoids and terpenes (Strongin, 2019).

To model CC vaping in the laboratory, it is necessary to understand user preferences, including the differences between CC and tobacco product vaping. Daily CC vaping, on average, is significantly less frequent (up to 10-fold less) compared to tobacco e-cigarette usage (Vreeke et al., 2022). This must be considered in computing toxicological risk assessments. However, there is a general lack of information concerning user topography (Morgan et al., 2022) in the context of CC inhalation. A recent survey of semisynthetic cannabinoid users (*N* = 267) showed a wide variety of user-reported vape temperatures (Bone et al., 2024). In addition, “chain-hitting” and ‘‘blinkers’’— vaping approaches that rely on built-in battery-cutoff mechanisms to auto-terminate vaporization rather than relying on the consumer to end inhalation—can reflect extensive puff duration times and vapor pressures that may impact temperatures relevant to promoting chemical reactions (Bone et al., 2024). In addition, survey responses from users, including preferences for vaping/dabbing as ‘‘hot as possible,” infer habits that elevate risk and reveal the need to better understand the differences in topography and related consumption habits within the vaping community (Bone et al., 2024).

### Terpene vaping chemistry

Terpenes have been of interest in the atmospheric chemistry field for decades (Atkinson, 1990). However, prior to our 2017 publication on terpene dabbing (Meehan-Atrash et al., 2017), there was a lack of information concerning terpene chemistry associated with vaping. To determine the contribution of terpenes towards the production of toxicant emissions, we investigated three prevalent terpenes found in cannabis and CCs: myrcene, linalool and limonene, along with a commercial terpene mixture for use in CC formulations. At the time of the study, crème brûlée torches were popular for heating the surface (“nail”) containing the sample. In addition, an informal search of online forums prior to our experimental work in 2016-2017 revealed the use of aerosolization temperatures between 340°C and 482°C (Meehan-Atrash et al., 2017).

Heating nail surface temperatures were monitored using a thermographic camera. The aerosols were passed through a cold trap and an impinger containing nuclear magnetic resonance (NMR) solvent, pulled by a vacuum from a commercial smoking machine. Alternatively, gas-phase molecules were collected on an adsorption thermal desorption (ATD) cartridge instead of an impinger for non-targeted analysis by automated adsorption–thermal desorption–gas chromatography–mass spectrometry (ATD-GC-MS), interfaced with the National Institute of Standards and Technology (NIST) database. Because dabbing topography had not been previously investigated, we chose an inhalation volume of 338 mL and a 10 s duration to assure a relatively complete collection of aerosol contents (Meehan-Atrash et al., 2017).

**FIGURE 1:**
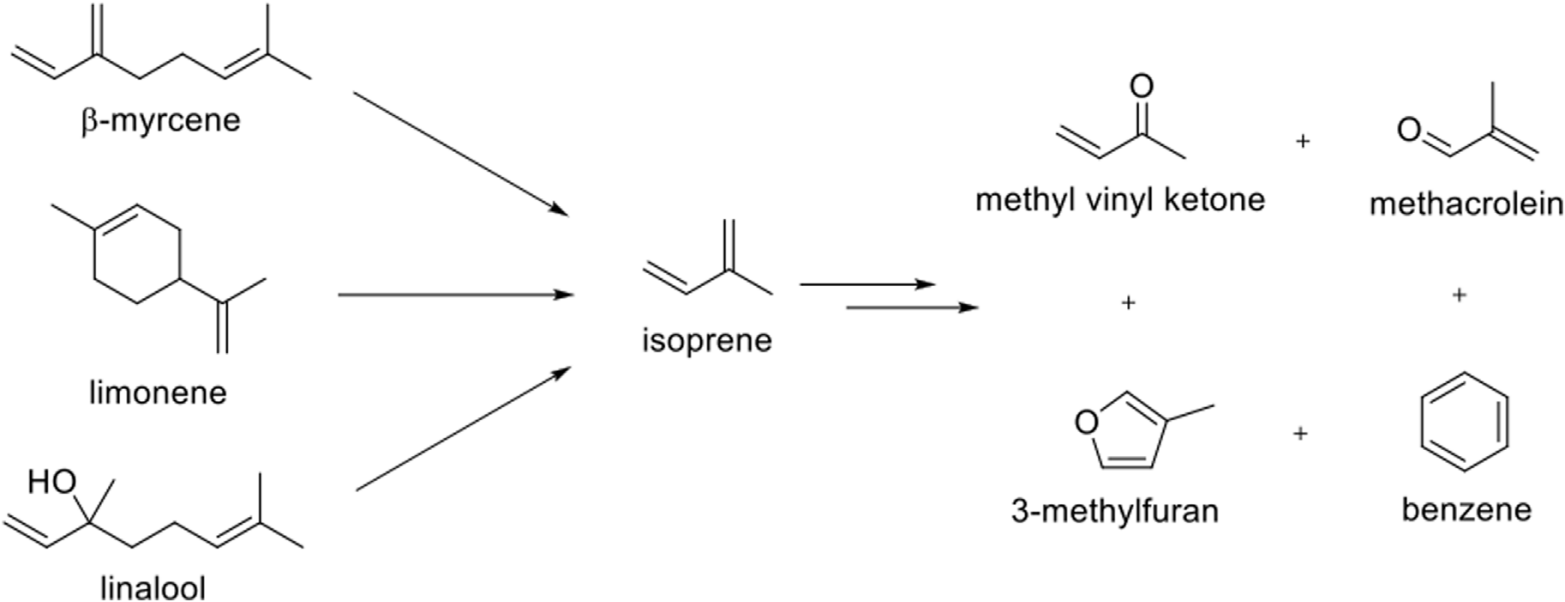
Select terpene degradation products identified via ATD-GC-MS analysis. These and numerous other related products were produced from pure samples of each of limonene, linalool, and myrcene. The products observed are consistent with terpene atmospheric chemistry. Adapted from reference (Meehan-Atrash et al., 2017).

Products from the reactions of each of the pure terpenes were consistent with those observed by atmospheric chemists probing the reactions of isoprene with hydroxyl radicals (Figure 1). (Atkinson, 1990) These included methyl vinyl ketone, methacrolein and 3-methylfuran (formaldehyde was not monitored in this study). Isoprene was also detected, as was benzene, but the latter only at the highest temperature used. Methacrolein, a well-known degradation product of isoprene (Atkinson, 1990; Carter, 1996; Palen et al., 1992), was readily observed by ^1^H NMR as well as by ATD-GC-MS. The concentrations of methacrolein per 40 mg dab were 185 ± 11 ppb at 526°C, 157 ± 2 ppb at 455°C, 131 ± 9 ppb at 403°C, and undetectable at 322°C. In addition to methacrolein and benzene, the ^1^H NMR spectra from the dabbing samples displayed numerous peaks, including those characteristic of organic acids, aldehydes, and aromatics (Meehan-Atrash et al., 2017).

These results are consistent with the prior terpene chemistry literature. For example, isoprene is a known degradation product of myrcene (Palen et al., 1992) and other terpenes (Britt et al., 2001). Benzene, alkyl benzenes, and polycyclic aromatic hydrocarbons are also known to form during terpene thermolysis. Benzene has previously been observed as a degradation product during the synthesis of myrcene via pyrolysis of β-pinene (Kolicheski et al., 2007), and as a product of solanesol pyrolysis (Britt et al., 2001). The gas chromatography-mass spectrometry (GC–MS) spectra of limonene, linalool, and myrcene each contained significant peaks corresponding to isoprene, indicative that the terpenes degrade to their constituent isoprene monomers. Overall, the key findings from this initial study were that terpene heating and aerosolization resulted in products consistent with terpene chemistry literature, including observations from prior synthesis/interconversion efforts as well as from atmospheric reactions largely involving hydroxyl radicals (Atkinson, 1990). Tang et al. (2021) and others Niu and Zhu (2023), Zhu et al. (2022) have published related studies of terpenoids found in CCs that also demonstrated the reactivity of these compounds under conditions relevant to vaping.

### ∆^9^- THC and terpene vaping chemistry

In a subsequent study Δ^9^-THC and terpenes were investigated along with commercial vaporizers as well as with a dab set-up (Meehan-Atrash et al., 2019). Samples modeled typical high potency CCs. The goals included determining which toxic VOCs or HPHCs derived from THC and which derived from terpenes. HPHCs are Harmful and Potentially Harmful Constituents, the compounds contained in tobacco smoke or other aerosols whose emissions the Food and Drug Administration (FDA) has determined to carry human health risks (US Food and Drug Administration, 2012). Interestingly, the VOCs found in cannabis smoke are known and are qualitatively similar to those found in tobacco cigarette smoke (Graves et al., 2020; Eyal et al., 2024). However, it was not known at the start of this investigation how emissions from vaping would compare to those from cannabis smoking. The aerosol product identifications and levels were used to estimate hazard indices (HIs) and excess lifetime cancer risks (ELCRs) of vaping and dabbing compared to each other and to cannabis smoking. The focus on gas-phase (VOC) products included the fact that the gas phase emission fraction contains the majority of known HPHCs.

**FIGURE 2:**
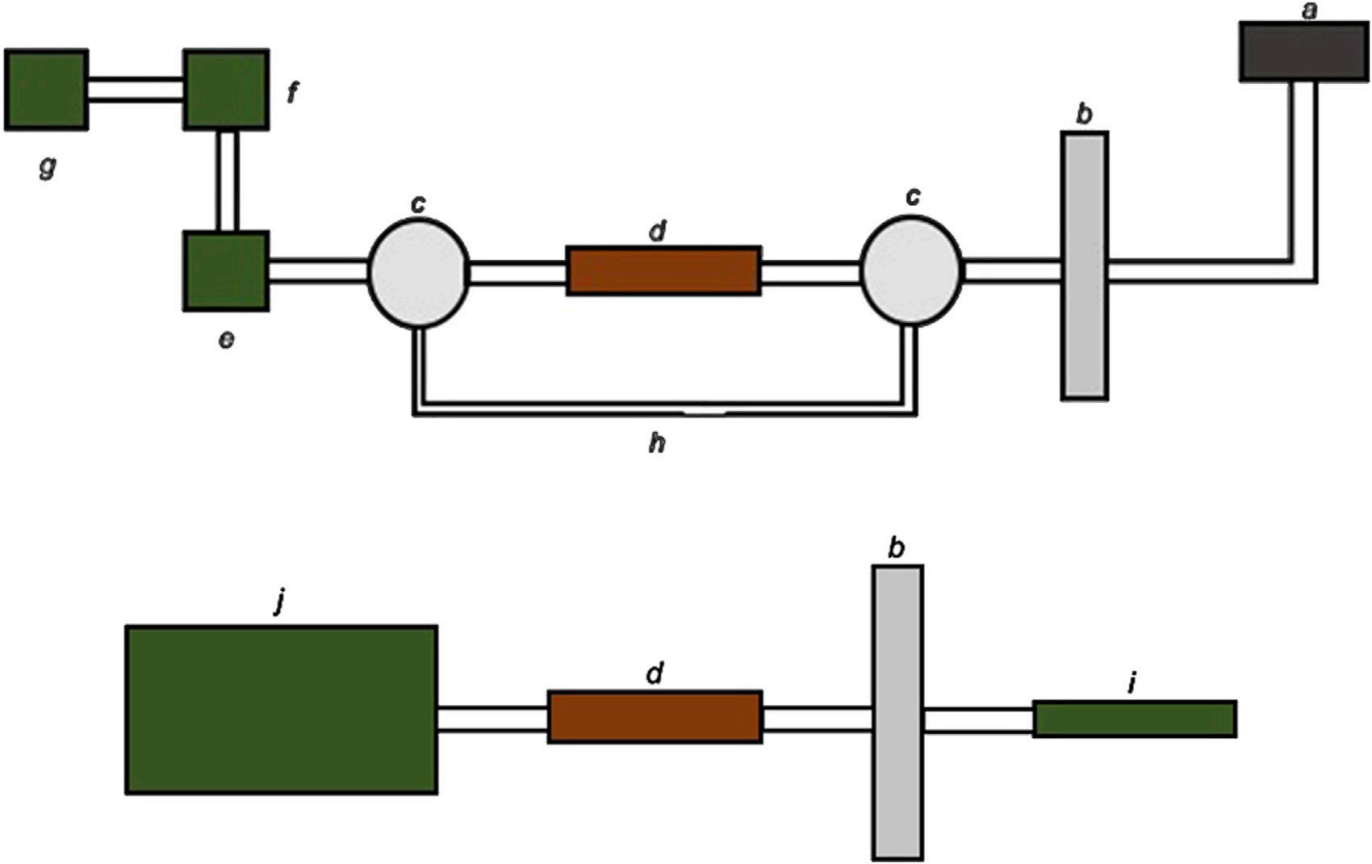
Experimental setups used for dabbing (top) and vaporizers (bottom) for aerosol VOC collection and analysis by ATD-GCMS. Components depicted are the (a) e-nail, (b) CFP holder, (c) three-way stopcock, (d) ATD cartridge, (e) mass flow meter, (f) flow control valve, (g) vacuum source, (h) by-pass line, (i) vaporizer, and (j) smoking machine. Used with permission from reference (Meehan-Atrash et al., 2019).

To enhance experimental control during dabbing, we replaced the smoking machine with a flow control valve, mass flow meter and a vacuum source, and added a by-pass line for additional control of the flow. In addition, the nail was replaced with an electronic nail (e-nail), affording more facile temperature regulation. For vaporizer investigations, we used a commercial CCELL device and an aerosol generation and collection setup that is essentially the same used for standardized tobacco e-cigarette studies (i.e., a modified Cooperation Centre for Scientific Research Relative to Tobacco, CORESTA protocol). The experimental vaping platforms used are diagrammed in Figure 2 (Meehan-Atrash et al., 2019).

**FIGURE 3:**
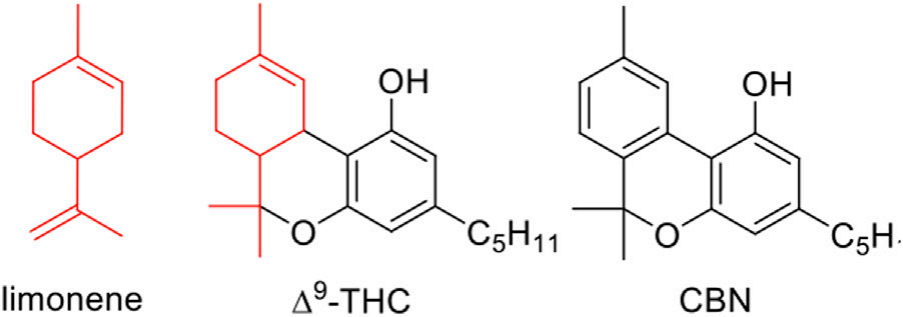
∆^9^-THC contains a reactive terpene substructure (red), whereas CBN contains an aromatic ring.

Table 1 (Meehan-Atrash et al., 2019) includes data corresponding to either a single 40 mg dab or to one puff from a vape pen. It is interesting that similar gas-phase compounds originate from terpenes and Δ^9^-THC. This is notable, as prevalent cannabinoids like THC and cannabidiol (CBD), among others, are terpenophenols featuring a cyclohexene substructure similar to that of limonene (Figure 3). Control tests using cannabinol (CBN), characterized by an aromatic ring instead of a cyclohexene-derived ring, produced significantly fewer and different products in comparison to THC and CBD, showing that most of the detected gas-phase products derive from the cyclohexene ring structure (Meehan-Atrash et al., 2019).

**TABLE 1:** Identities and levels of selected gas phase HPHCs found in aerosols. SND = synthetic distillate, 9:1 THC: terpenes.

Compound, unit	THC dab	SND dab	Vape 3.2 V	Vape 4.0 V	Vape 4.8 V
Methacrolein, μg	2.7 ± 0.8	12 ± 0.82	5.6 E−3	3.2 E−2	1.9 E−1
Benzene, ng	33 ± 14	360 ± 120	9.9 E−1	2.7 E+0	3.6 E+1
Xylenes, μg	0.33 ± 0.20	0.85 ± 0.30	1.0 E−3	1.5 E−2	1.8 E−1
Toluene, μg	0.44 ± 0.22	1.4 ± 0.42	7.0 E−4	1.0 E−2	1.6 E−1
Styrene, ng	0.88 ± 0.72	27 ± 14	9.3 E−2	2.7 E−1	ND*
Ethylbenzene, ng	1.5 ± 0.99	55 ± 30	3.7 E−2	2.5 E−1	2.7 E+0
Isoprene, μg	9.6 ± 1.7	44 ± 3.5	3.0 E−2	8.3 E−1	6.0 E+0
Other VOCs,^†^ μg	5.3 ± 0.7	21 ± 11	4.2 E−2	7.2 E−1	7.9 E+0
Total VOCs,^‡^ μg	2.0 E+01	7.7 E+01	9.4 E−2	1.5 E+0	1.2 E+1

For dabbing experiments, HPHCs, were quantified by internal standard calibration, and represent normalized levels from a (realistic) 40 mg dab ±SEM (duplicate runs). Isoprene levels in dabbing were estimated by an internal standard response factor analysis (IS-RF). Gas phase components found by vaping at the 3 voltages are from single puff measurements, estimated using IS-RF, analysis. *Styrene was not detected in CV, vaping at 4.8 V due to overlap of alkenic terpene degradation products (Figure 5).

^†^Non-targeted VOCs, not specified in this table.

^‡^Total of all VOCs, quantified. Used with permission from reference (Meehan-Atrash et al., 2019).

Although benzene, methacrolein, and isoprene—compounds present in smoke from burned cannabis cigarettes—were previously established to come from terpenes (Meehan-Atrash et al., 2017), the data in Table 1 shows they are also produced from Δ^9^-THC. Despite the relatively lower amounts of toxic VOCs in vaping emissions compared to combusted plant material smoke, they continue to be a source of concern. For example, cardiovascular health hazards may endure despite comparatively decreased toxicant doses over time (Pope et al., 2009; Bhatnagar, 2016). This is because the dose–response relationship between smoking and cardiovascular mortality is nonlinear. Most (80%) of the harm occurs at low doses of <3 cigarettes per day. Thus, a reduction in toxicant exposure from vaping devices will not result in proportional harm reduction. Furthermore, numerous gas-phase ATD-GC-MS chromatogram peaks were unidentifiable. The aerosol quantities in the particle phase, encompassing cannabinoids and their derivatives, were not monitored nor included in the computations. Notwithstanding these constraints, quantitative risk assessments were performed for the conditions specified in Table 1. A hazard index (HI), which evaluates non-cancer risk, and an excess lifetime cancer risk (ELCR), which estimates cancer risk over a lifetime, were computed and juxtaposed with risks linked to smoking (Table 2; Meehan-Atrash et al., 2019). The smoking data derived from a comprehensive literature study. Consistent with expectations, both ELCR and HI values escalated with smoking compared to vaping and with elevated power levels. This observation corresponds to the prevailing belief that vaping has fewer health risks than smoking. However, additional study is required to validate this belief. As noted above, significant limitations include the lack of particulate-phase data, the large quantity of unidentified gas-phase compounds, and dependence on many assumptions within the quantitative risk assessment methodology (Meehan-Atrash et al., 2019). Despite lower gas-phase toxicant yields from dabbing and vaping compared to smoking, there is a need for further research to understand the full health implications, especially given the relatively high concentrations of THC in CC aerosols as well as the potential for acute effects, such as observed during the EVALI outbreak (Rebuli et al., 2023).

**TABLE 2:** Hazard Index and excess lifetime cancer risk for smoking, dabbing, and vaping at three voltages.

Consumption type		HI	ELCR
Smoking (inflorescence)		2 × 10^2^	4 × 10^-4^
Dabbing (distillate)		2 × 10^-1^	2 × 10^-7^
Vaping	4.8 V (distillate)	4 × 10^-2^	2 × 10^-7^
	4.0 V (distillate)	6 × 10^-3^	2 × 10^-8^
	3.2 V (distillate)	8 × 10^-4^	2 × 10^-9^

HI, and ELCR, calculations assumed the consumption of one 0.75 g joint, two 40 mg dab, and 20 puffs from a vape pen for each voltage. Quantitative risk assessment has several unavoidable sources of uncertainty, which is magnified herein due to the lack of standardization in the study of cannabis consumption as compared to tobacco and other limitations noted in the text. An HI, value of ≤ 1 means the exposure is not likely to cause adverse non-cancer health effects. An ELCR, value ≤10^-6^ is typically considered acceptable, with a range of 10^−6^-10^-4^ considered acceptable in some cases. Several unidentified compounds were identified in reference 36 that lacked chronic toxicity values precluding their risk estimation, adding to the overall uncertainty in the risk estimates (and likely bias towards underestimation). Used with permission from reference (Meehan-Atrash et al., 2019).

### Mechanistic studies

**SCHEME 1:**
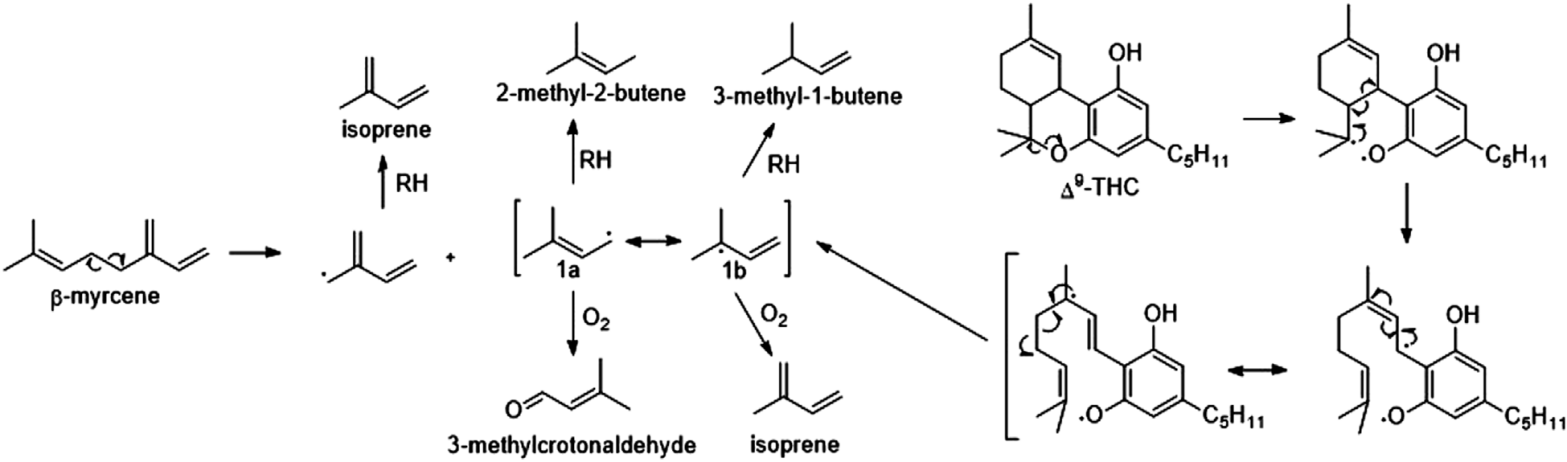
Proposed mechanisms for the thermal degradation of ẞ-myrcene and Ao-THC under vaping (heat and aerosolization) conditions. A°-THC reacts to form the same intermediate (1a/1b) as myrcene, thereby accounting for the same four products from each of A'-THC and myrcene. Myrcene is the most prevalent terpene found in cannabis. The four products, 2-methyl-2-butene, 3-methyl-1-butene, 3-methylcrotonaldehyde and isoprene comprise 30% of the total VOCs derived from myrcene and 22 % of the VOCs from ^'-THC. The relative distributions of the four products vary with temperature. Adapted from reference (Meehan-Atrash et al., 2017).

A subsequent mechanism-focused investigation began with a rigorous study of the gas-phase products formed upon dabbing pure myrcene and pure ∆^9^-THC samples (Meehan-Atrash et al., 2021) Of the numerous product peaks displayed in the ATD-GC-MS data, and based on isotopic labeling of myrcene, we proposed a reaction mechanism to account for a significant percentage of the total aerosol VOCs from myrcene and from ∆^9^-THC (Scheme 1). Four products—3-methylcrotonaldehyde, 2-methyl-2-butene, isoprene, and 3-methyl-1-butene—were identified as accounting for 30% and 22% of the total gas-phase products from myrcene and ∆^9^-THC vaping, respectively. There is a lack of toxicological data for 3-methylcrotonaldehyde. However, according to the Agency for the Study of Toxic Substances and Disease Registry (ASTDR), crotonaldehyde is highly toxic when inhaled. Exposure causes inflammation and irritation of the skin, respiratory tract, and mucous membranes. Delayed pulmonary edema may occur after inhalation. (ASTDR. Available online at) Isoprene can cause respiratory irritation and act as a central nervous system (CNS) depressant and is an asphyxiant at high concentrations. It is classified as a reasonably anticipated to be a human carcinogen (Group 2B) (National Toxicology Program, 2011). 2-Methyl-2-butene and 3-methyl-1-butene are known petroleum distillates and components of gasoline that are aspiration hazards and may cause pulmonary damage, central nervous system depression, and cardiac arrhythmias. They may also affect the blood, immune system, liver, and kidney (ASTDR, 2012).

In a 95:5 Δ^9^-THC:myrcene mixture, the inclusion of myrcene afforded a 5-fold increase in isoprene emission compared to using 100% Δ^9^-THC. At 90:10 Δ^9^-THC:myrcene, the additional myrcene concentration resulted in a more than sixfold increase in isoprene levels. These findings correspond to (i) an increased reactivity of terpenes relative to THC and (ii) myrcene’s propensity to partition into the gas phase, rendering it more susceptible to gas-phase interactions (Meehan-Atrash et al., 2021).

However, in experiments using a CCELL vaporizer, rather than the dabbing platform, increasing the myrcene:THC proportion led to lower yields of isoprene and other VOC degradation products, the opposite trend observed during dabbing (Meehan-Atrash et al., 2021). This discrepancy in the trends of degradant formation based on increasing myrcene levels in each of the two routes of administration can be explained by the fact that terpenes reduce THC viscosity. As noted previously, viscosity reduction improves heat transfer efficiency via enhancing wicking efficiency in vaporizers—a critical factor in reducing aerosol yields of toxic VOCs in e-cigarettes whereas dabbing, which does not require wicking, is more impervious to viscosity effects.

In summary, the relative ratios of two primary components of CCs, Δ^9^-THC and myrcene, affect their dosage as well as exposure to HPHCs and other toxic VOCs (Meehan-Atrash et al., 2021). Continued research into the chemical and physical factors influencing aerosol toxicant profiles will aid in developing safer cannabis products that minimize health risks and optimize harm reduction. However, the next sections include cautionary tales concerning additives as well as the chemical manipulation of a cannabinoid that leads to the formation of one of the most toxic vaping emissions currently known.

### Additives

Cases of e-cigarette–related respiratory disorders have been well-documented from 2012–present (Rebuli et al., 2023). In 2019, e-cigarette or vaping product use–associated lung injury (EVALI) emerged as an epidemic (Perrine et al., 2019). During mid-2019 through February of 2020, when the CDC was monitoring US cases, 2,807 individuals were hospitalized nationwide with EVALI and 68 patients died (Rebuli et al., 2023). Although there are ongoing reports of EVALI hospital admissions, there has been a significant decline in patient numbers since 2020. (Rebuli et al., 2023) The decline coincided with the onset of the COVID-19 pandemic in 2020, along with the discovery of vitamin E acetate (VEA) as a chemical strongly associated with EVALI. Although VEA was found in a relatively large percentage of EVALI patient samples (Ashley et al., 2020), the underlying pathophysiological mechanism of EVALI currently remains unknown (Rebuli et al., 2023).

During the EVALI epidemic our lab received two unknown samples that we were informed were being used in CC formulations. One of the vials contained VEA. The other vial included mainly pine rosin and medium chain triglycerides (MCT oil, a solvent) as well as a hypnotic agent, oleamide (Table 3; Meehan-Atrash and Strongin, 2020). Oleamide is a relatively common additive used in illegal synthetic marijuana (colloquially “Spice” or “K2”) (Gunderson et al., 2012). We were unaware of oleamide or pine rosin having been used previously in any CC formulations. Pine rosin is a readily available substance utilized in industrial varnishes, adhesives, and sealing wax. It helps musicians and athletes grip bowed string instruments and athletic equipment (Sarria-Villa et al., 2021). Pine rosin is an FDA-approved food additive; however, it is a respiratory tract irritant and a major contributor to occupational asthma caused by its presence in solder fumes (Tiotiu et al., 2020).

**TABLE 3:** Components of a sample containing pine rosin (diterpenoids, abietic and other resin acids) along with MCT oil and oleamide. Compounds were identified by NMR and HPLC-ESI-MS. Approximate percentages in the sample were determined by Q-NMR. Used with permission from reference (Ashley et al. (2020)).

Common Name	CAS Number	RT in LC/MS (min.)	NMR Shift (ppm)	Mass Accuracy (ppm)	% in Sample
Dehydroabietic acid	1740-19-8	16.5	6.88	0.03	3
Communic acid	2761-77-5	21.8	6.32	0.03	4
Pimarol	1686-59-5	23.9	NA	0.52	NA
Pimaric acid	127-27-5	23.9	5.71	1.25	3.2
Sandaracopimaric acid	471-74-9	23.9	5.22	1.25	1.5
Palustric acid	1945-53-5	23.9	5.39	1.25	14
Abietic acid	514-10-3	25.1	5.77	1.25	17
Oleamide	301-02-0	25.1	6.65-7.19	0.64	NA
Neoabietic acid	471-77-2	25.1	6.2	1.25	12
Isopimaric acid	5835-26-7	25.1	5.81	1.25	13
Sandaracopimarinal	3855-14-9	30.3	5.22	0	NA
MCT oil	438544-49-1	NA	4.3	NA	15

**FIGURE 4:**
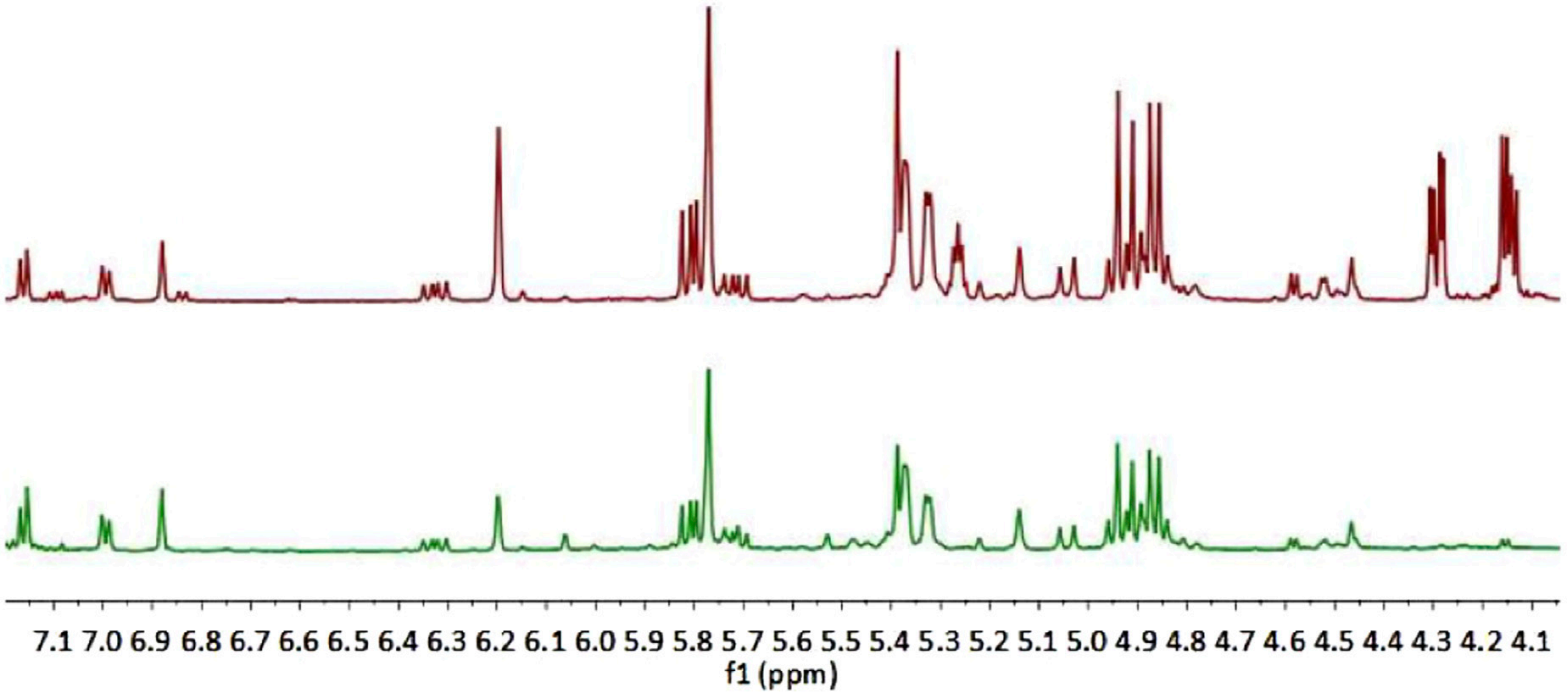
Overlay of the 'H NMR spectrum of the unknown sample (top, maroon) and a commercial sample of gum rosin (bottom, green). See Table 3 for key NMR peak assignments. Used with permission from reference (Meehan-Atrash and Strongin, 2020).

It was relatively straightforward to identify pine rosin in the sample via the ^1^H NMR spectrum overlay with that of an authentic commercial sample (Figure 4; Meehan-Atrash and Strongin, 2020). When incorporated into a CC at a concentration of only 1%, approximately 0.6 g/m^3^ of pine rosin can be transferred into the aerosol from a cannabis vape pen with each inhalation, equating to roughly 3,500 times the 15 min time-weighted average exposure limit (Baldwin et al., 2007). *In vivo* exposure to abietic acid, the main component of pine rosin, to rat lungs, resulted in desquamation of the bronchial epithelium, a phenomenon also documented in EVALI cases (Butt et al., 2019). Following our report, FDA scientists reported finding pine rosin in a study including EVALI patient samples (Ciolino et al., 2021).

### Ketene from cannabinoid and other acetates

**FIGURE 5:**
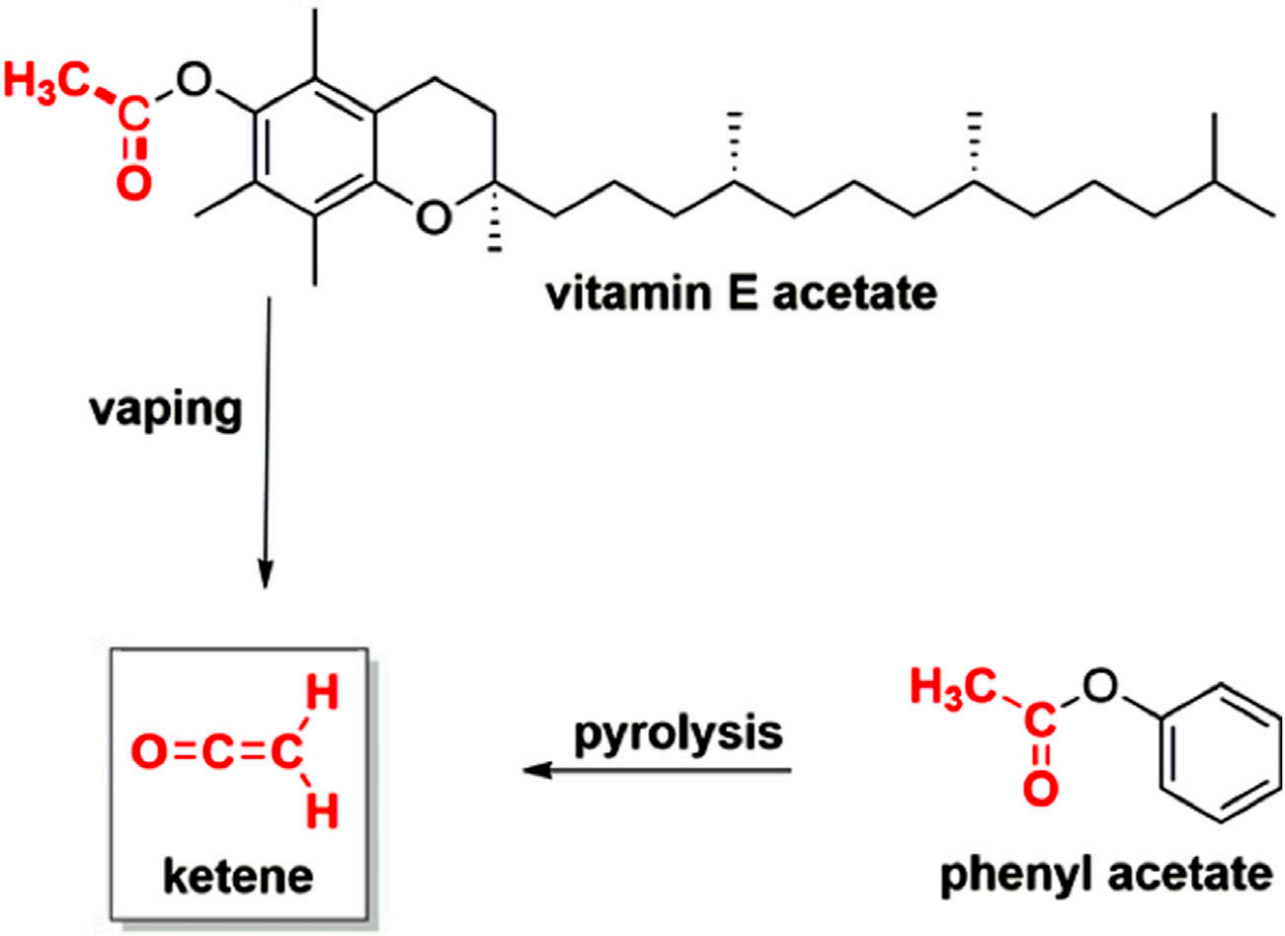
The pyrolysis reaction of phenyl acetate to produce ketene was reported in 1938. The analogous transformation of vitamin E acetate to ketene was shown in 2020 to occur upon heating and aerosolization in a commercial vaping device. Adapted from reference (Strongin, 2019).

After an association between VEA and EVALI was established (Blount et al., 2019; Duffy et al., 2020; Ellington et al., 2020), there were numerous investigations of the ability of VEA to chemically degrade upon heating and aerosolization to produce toxic emissions (Li et al., 2022; Canchola et al., 2023; Canchola et al., 2022; LeBouf et al., 2022; Kovach et al., 2022; Alcon, 2022; Jiang et al., 2020; Xantus et al., 2021; Kosarac et al., 2021; Lynch et al., 2021; Borgonhi et al., 2021; Mikheev and Ivanov, 2022). Of these studies, one of the most significant was reported by Wu and O’Shea who showed that ketene is emitted upon vaping VEA in a commercial vaporizer (Wu and O'Shea, 2020) VEA contains a phenyl acetate substructure. Phenyl acetate has been known for nearly a century to produce ketene upon thermal decomposition (Figure 5) (Hurd and Blunck, 1938).

Ketene (CH_2_=C=O) is a poisonous gas that presents considerable health hazards, even at relatively minimal doses. The Acute Exposure Guideline Levels (AEGL)-3 (life-threatening levels) for ketene are 0.24 ppm (0.41 mg/m^3^) for 10 min and 0.088 ppm (0.15 mg/m^3^) for 8 h (Council, 2010). The biochemical and toxicological properties of ketene mirror those of phosgene (Cl_2_C=O), a WWI chemical warfare agent, as a reactive acylating agent and respiratory poison. Ketene is also genotoxic. (EPA, 2025) Ketene is a powerful electrophile that exhibits high reactivity with tissues containing proteins and nucleic acids. Prolonged exposure to low concentrations of ketene may result in cumulative pulmonary damage and respiratory issues; however, chronic exposure has not been extensively investigated (Council, 2010). Wu and O’Shea noted similarities in the symptoms of acute ketene exposure to those presented by EVALI patients (Wu and O'Shea, 2020).

**FIGURE 6:**
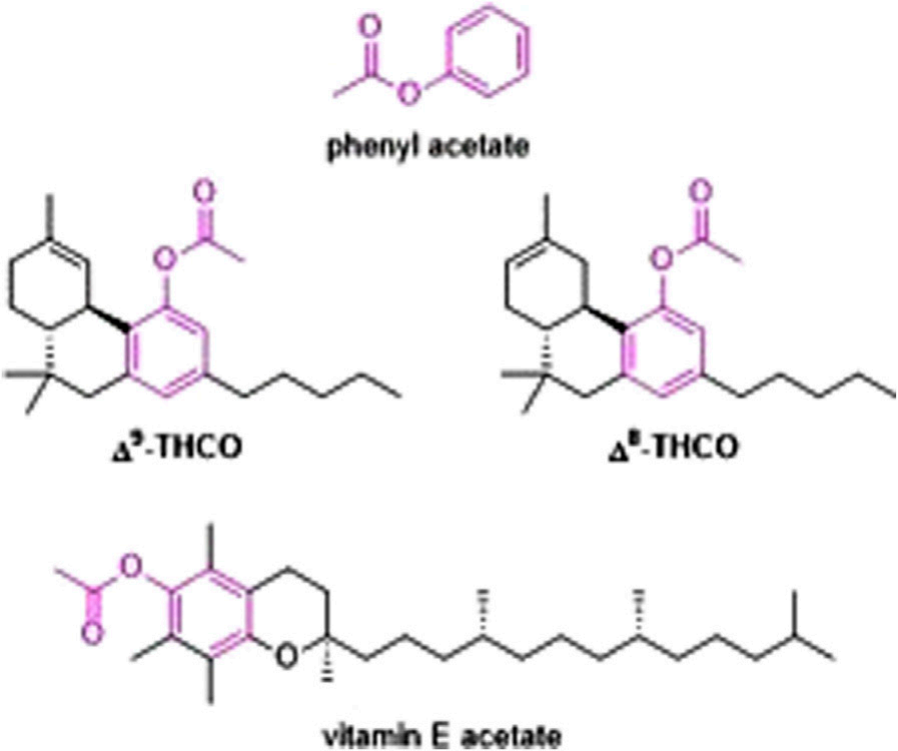
Cannabinoid acetates possess a phenyl acetate substructure, the same source of ketene emissions observed upon vaping vitamin E acetate (Figure 5).

Given the toxicity of ketene, and the fact that it could be produced upon vaping VEA in an e-cigarette, we were surprised and concerned to find that ∆^8^-THC acetate (∆^8^-THCO) emerged as a commercially available product. Our concern stemmed from the fact that THC acetates possess the same phenyl acetate substructure as VEA, as illustrated in Figure 6. Moreover, due to the 2018 Farm Bill (Abernethy, 2019), ∆^8^-THC, despite having psychoactive properties, was, legal throughout the US when we began this investigation. However, ∆^8^-THC is not as potent as Δ^9^-THC (Tagen and Klumpers, 2022), which is likely why ∆^8^-THC is acetylated to THCO. Acetylation can enhance a compound’s psychoactivity by enabling it to cross the blood-brain barrier more readily due to a reduction in molecular polarity. For example, heroin is acetylated morphine (Munger et al., 2022) We indeed found that ∆^8^-THCO, and all other model cannabinoid acetates tested (Δ^9^-THCO, CBNO and CBDO, as well as a pre-filled commercial ∆^8^-THCO cartridge vape pen), each produced ketene emissions under real-world vaping conditions, including at levels in range of the National Institute for Occupational Safety and Health (NIOSH) thresholds; (Munger et al., 2022) e.g;., 8-h Time Weighted Average (TWA) 0.5 ppm (0.9 mg/m^3^), and/or 15 min Short Term Exposure Limit (STEL) 1.5 ppm (3 mg/m^3^). These worker protection standards are >5-fold higher than the AEGLs described herein. Soon after our report, Benowitz et al. independently showed that vaping a commercial sample containing ∆^8^-THCO from a different vendor than our sample, also led to ketene emission (Benowitz et al., 2023).

**FIGURE 7:**
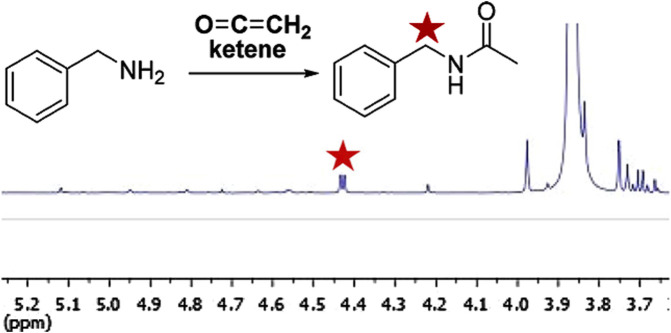
^1^H NMR spectrum expansion of a vaped sample of CBNO trapped by benzylamine in an impinger containing CDCl3. The star indicates the doublet (4.43 ppm) corresponding to the N- benzylacetamide methylene. Reprinted with permission from reference 86. Copyright 2025 American Chemical Society.

Due to its extreme reactivity, ketene detection is based on a reaction with a nucleophilic trapping agent such as benzylamine (Wu and O'Shea, 2020). The resultant *N-*benzylacetamide product can be characterized and quantified by NMR and/or chromatographic techniques. Figure 7 shows the ^1^H NMR spectrum of a vaped sample of CBNO after ketene trapping by benzylamine in an impinger containing CDCl_3_ (Munger et al., 2022).

Using benzylamine as a trapping agent, however, may not be optimal since amines are relatively nonselective reagents. Benzylamine use for ketene trapping and determination should thus ideally be limited to relatively well-defined systems that do not contain molecules (such as other acetates) that can react to form the same *N*-benzylacetamide product as ketene. However, manufacturers are not required to disclose most vaping product ingredients, and moreover, heating and vaping produce aerosols containing complex chemical mixtures.

The reaction in the impinger at rt between ketene and benzylamine is complete within minutes. To justify ketene selectivity, we hypothesized that the reaction of benzylamine with potential interferents (other acetates) to give *N*-benzylacetamide would be much slower. Indeed, when unvaped CBNO or ethyl acetate was stirred for 8 h in the same impinger solution used to trap ketene after vaping, either no or trace product formation (respectively) was observed by ^1^H NMR (Munger et al., 2022). This is encouraging evidence of kinetic selectivity for ketene.

**SCHEME 2:**
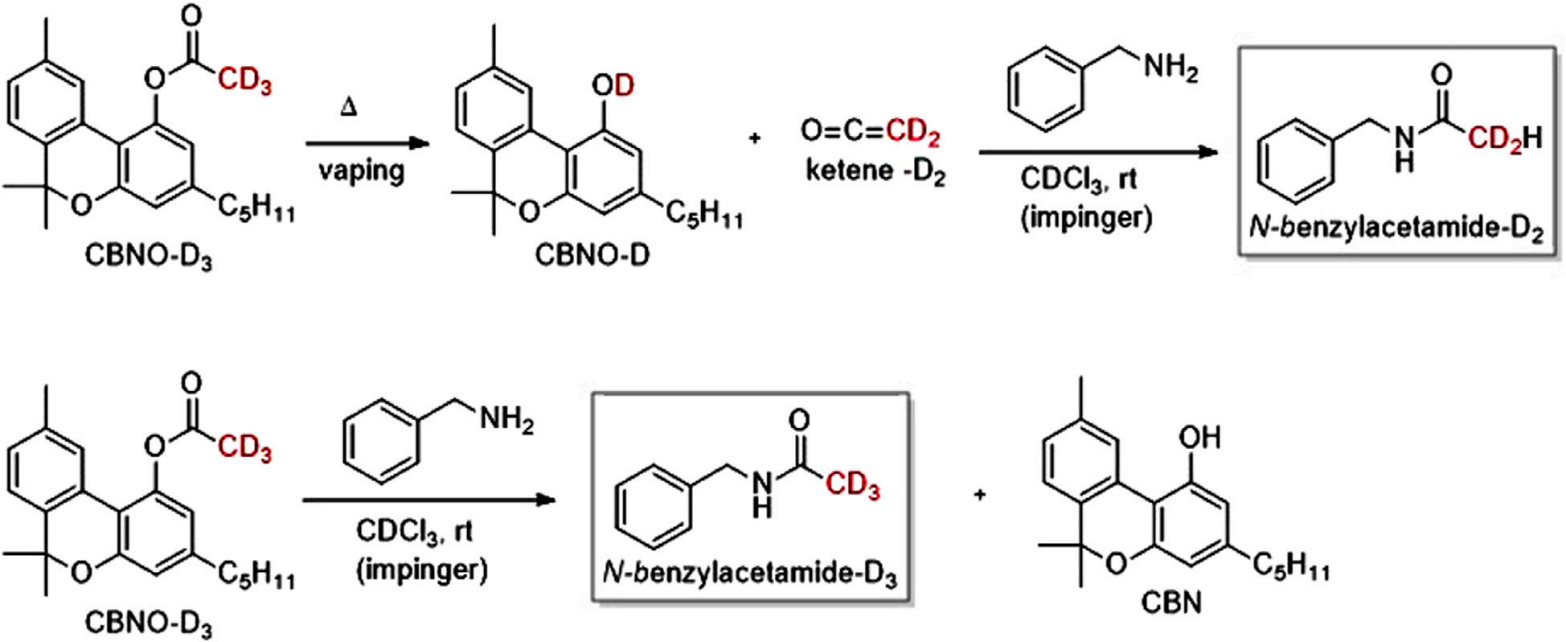
Top: A trideuterated acetate must lose a deuterium to form ketene, therefore resulting in an N-benzylacetamide-D2H product. Bottom: Alternatively, N-benzylacetamide-D3 will be the product if a different (e.g., addition-elimination) mechanism not involving a ketene intermediate is relevant. Figure 8 shows 13C NMR evidence for N-benzylacetamide-D2 from CBN-OAc-D2H via ketene. Used with permission from reference (Munger et al., 2024).

To more rigorously identify ketene’s origin and formation during vaping we synthesized isotopically labeled CBNO-D_3_. The main hypothesis addressed via isotopic labeling is illustrated in Scheme 2. The top Scheme shows that a dideuterated *N*-benzylacetamide product (*N*-benzylacetamide-D_2_H) would result if a ketene intermediate is formed from the starting trideuterated acetate methyl (i.e., CBN-OAc-D_3_). Conversely (bottom), if *N*-benzylacetamide-D_3_ is observed after vaping CBNO-D_3_ then ketene was not a reactant, and likely the common addition-elimination reaction pathway involving a direct reaction of CBNO-D_3_ and benzylamine occurred in the impinger without ketene intermediacy.

**FIGURE 8:**
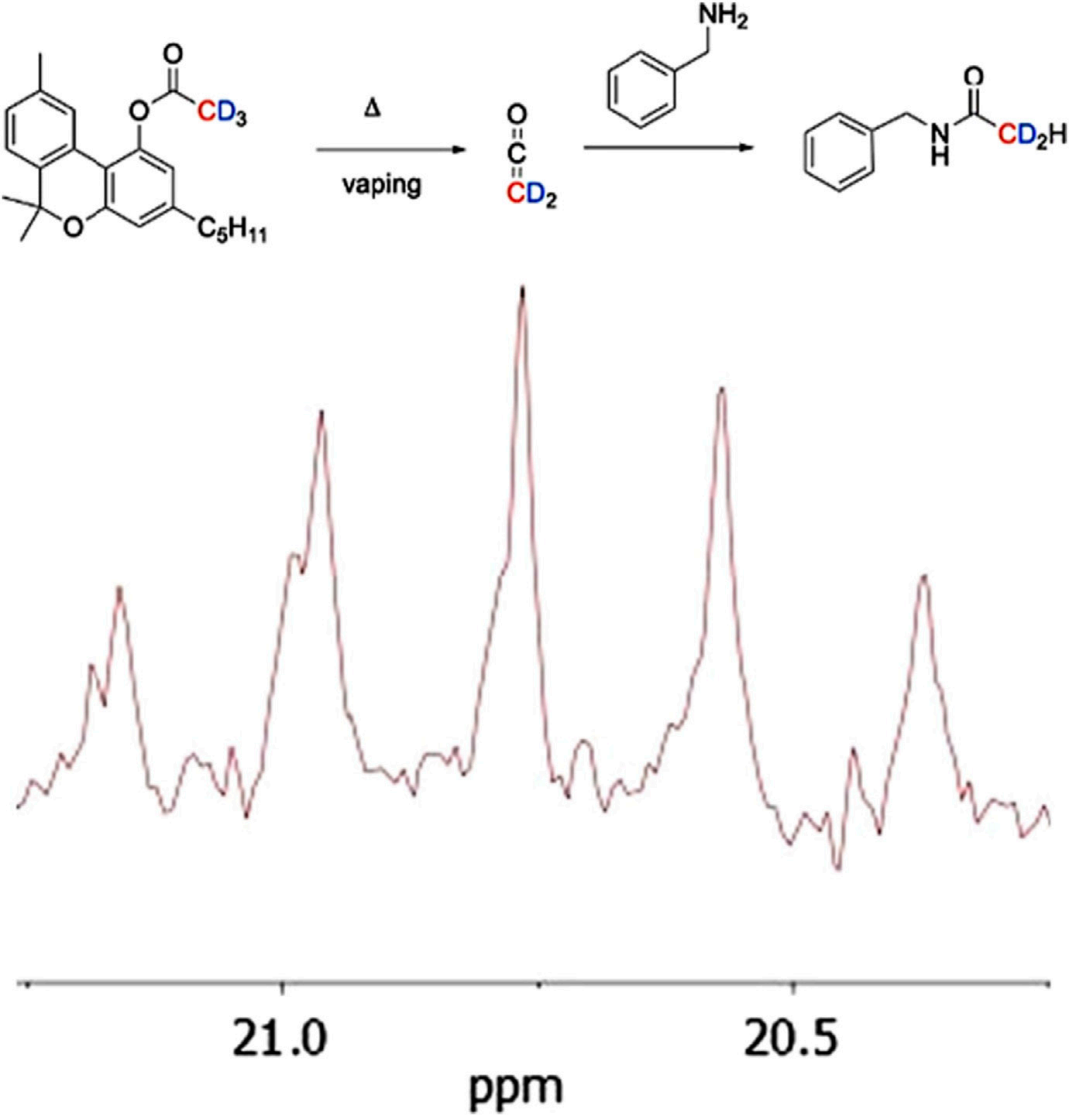
Expansion of the 13C NMR spectrum (CDCl3) of the aerosol generated under flash vaporization conditions (378 °C) on a quartz surface showing the anticipated quintet splitting pattern (J= 20 Hz) corresponding to the dideuterated acetate methyl carbon of N-benzylacetamide-D_2_. Used with permission from reference 96.

Vaping CBNO-D_3_ led to the data in Figure 8 showing the expansion of the ^13^C NMR spectral region where the acetate methyl carbon resonance of *N*-benzylacetamide is found. The pentet splitting pattern is clearly indicative of a dideuterated carbon; i.e., the product *N*-benzylacetamide-D_2_H formed via a ketene intermediate.

It is important to keep vaping experimental temperatures in line with realistic usage. During the course of this study in 2023-2024, we collaborated on a peer-reviewed systematic survey and analysis of user-preferred THC-acetate vaporizer and dab platform temperatures (Bone et al., 2024). Temperatures ≥378°C, which we had been using in the studies described so far in this section were preferred by 8% of respondents. For relevance to a wider range of users, we thus investigated and found readily detectable levels of ketene equivalents as *N*-benzylacetamide when the aerosolization was performed at 250°C, a temperature setting at or above which >70% of the survey respondents reported dabbing and 40% of respondents reported vaping (Bone et al., 2024). We determined that a 40 mg dab of CBNO at 250°C furnished a 0.005 mg yield of ketene as *N*-benzylacetamide equivalents (Munger et al., 2022). Considering the 22% trapping efficiency of ketene by benzylamine (Munger et al., 2022), the actual emission yield of ketene from the single puff is likely closer to ∼5-fold higher (∼0.025 mg) and thus within an order of magnitude of the Acute Exposure Guideline Levels (AEGL-3 life-threatening levels, 10 min).

More recently, Wang et al. studied conditions leading to the production of ketene during VEA vaping (Wang et al., 2024). They also concluded that, in the case of both VEA and THCO, ketene can be produced under realistic vaping temperature conditions. They also used a unique method to profile heating filament temperatures, and found that vaping VEA led to filament temperatures as high as 510°C. They noted significant discrepancies between user-reported vaping temperatures and actual values, including large fluctuations in vape temperature readings (Wang et al., 2024).

The issue of temperature is especially relevant in the case of ketene formation during vaping. According to recent theoretical research, any significant amounts of ketene must develop from VEA or related acetates at unreasonably high vaping temperatures—above at least 700°C (Benowitz et al., 2023; Narimani and da Silva, 2020; Narimani et al., 2022). Some have attributed potential catalysis or inefficient wicking as the source of ketene generation at comparatively low temperature levels (Benowitz et al., 2023). However, the simulations were performed in an oxygen-free model system.

We propose that oxygen is a significant contributor to ketene production at real-world vaping temperatures. This is based on earlier research that our group published in 2017 (Jensen et al., 2017), showing that a major chemical process in e-cigarette vaping leading to toxic emissions is oxidation. Marked decreases in vaped aerosol toxicant concentrations in a reduced oxygen atmosphere were observed by us. (Jensen et al., 2017) Subsequent studies by us and other researchers (Canchola et al., 2023; Korzun et al., 2018; Jaegers et al., 2021) have confirmed that oxygen overall promotes chemical breakdown in the case of both nicotine and cannabis vaping. The results are also in line with research by Son et al. and (Canchola et al., 2023) that demonstrated the production of hydroxyl radicals during vaping.

**FIGURE 9:**
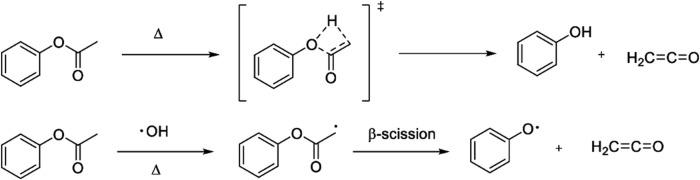
Two mechanisms for formation of ketene from phenyl acetate. Top: the published concerted four-membered ring transition state. Bottom: mechanism involving oxygen-derived hydroxyl radicals analogous to that previously described for ketene formation from the combustion of small aliphatic acetates such as ethyl, methyl, and isopropyl acetate. Used with permission from reference (Munger et al., 2024).

Figure 9 shows the mechanism of formation of ketene from phenyl acetate via a four-membered ring transition state as proposed by Nishida et al., in 1974 (Nishida et al., 1974), along with our current proposed mechanism (Munger et al., 2024) showing a potential role for hydroxyl radicals. The latter mechanism is precedented for acetates, adapted from earlier published acetate combustion studies (Hoare and Kamil, 1970; Lam et al., 2012). It involves proton abstraction by hydroxyl radical followed by β-scission to afford ketene. Importantly, as noted above, radicals (Bitzer et al., 2018a; Bitzer et al., 2018b), including hydroxyl radicals (Canchola et al., 2023; Son et al., 2019), are prevalent during vaping.

**FIGURE 10:**
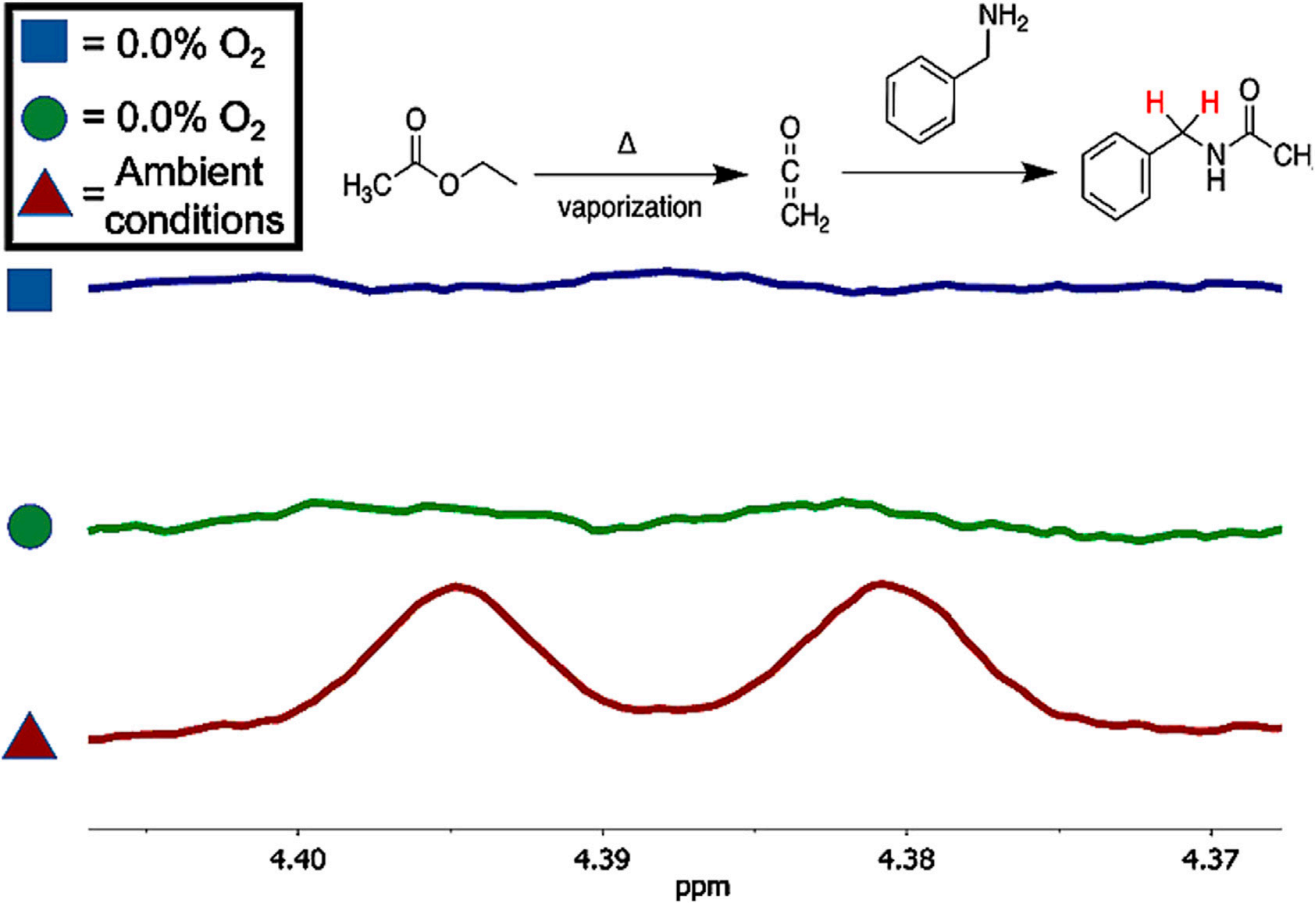
Expanded 'H NMR spectra showing the methylene protons of N-benzylamide that was formed upon collection of the aerosol generated by ethyl acetate under ambient atmospheric conditions (red, bottom spectrum) along with two trials conducted in a 0.0% O2 (<LOD of instrument) atmosphere, displayed in the green (middle) and blue (top) spectra. qNMR analysis of the samples was conducted and shows that ambient atmospheric conditions generated ten times the amount of ketene (trapped as N- benzylacetamide) when compared to the ketene generated under anaerobic conditions. Used with permission from reference (Munger et al., 2024).

Importantly, recent studies show that ketene can originate from heating and aerosolizing compounds besides VEA and cannabinoid acetates, and that the mechanism involves oxygen (Munger et al., 2024). Figure 10 highlights the differences in ketene emissions in based on vaping ethyl acetate or geranyl acetate in an ambient atmosphere versus in a deoxygenated atmosphere. Note one study showed that ethyl acetate is the fifth most frequently occurring flavor chemical in a study of 277 commercial Electronic Nicotine Delivery System (ENDS) e-liquids: esters are the most prevalent class of ENDS flavorants (Omaiye et al., 2019). Geranyl acetate is a common terpenoid found in many cannabis formulations: terpenes are the second most abundant class of ENDS flavorants: (Omaiye et al., 2019)

## Summary and conclusion

Early studies on cannabis vaping found aldehyde emissions when aerosolizing BHO mixed with propylene glycol, but the contribution of solvents to toxicant formation was unclear. To better model concentrate (CC) vaping, a 9:1 THC:terpene mixture was developed to balance viscosity for vaporization without added solvents. Using dabbing methods allowed differentiation of chemical reactivity effects from viscosity-related toxicant formation.

Prior to 2017, little was known about cannabis terpene chemistry in vaping contexts. Aerosolization of myrcene, linalool, limonene, and a commercial terpene mixture using dabbing methods revealed toxic degradation products consistent with atmospheric chemistry literature, including methacrolein, isoprene, benzene, and various aldehydes and acids. These findings confirmed that terpene heating during vaping generates toxicants through mechanisms similar to known atmospheric and pyrolytic reactions.

Toxic gas-phase emissions from vaping and dabbing high-potency cannabis concentrates (CCs) using both dab rigs and commercial vaporizers were investigated to distinguish harmful and potentially harmful constituents (HPHCs) originating from Δ^9^-THC versus terpenes, and to compare emissions to those from cannabis smoking. Results showed that both Δ^9^-THC and terpenes produced similar volatile organic compounds (VOCs), including benzene, methacrolein, and isoprene, primarily linked to their cyclohexene structures. Although vaping and dabbing produced fewer toxicants than smoking, cardiovascular concerns remain due to nonlinear dose–response effects. Quantitative risk assessments (hazard index and excess lifetime cancer risk) confirmed lower risk for vaping relative to smoking, but significant unknowns and limitations highlight the need for further research.

Adding myrcene to pure Δ^9^-THC increased isoprene formation during dabbing, but not during vaping with CCELL devices, due to terpene-driven modulation of viscosity effects and heat transfer. These findings show that Δ^9^-THC:terpene ratios critically influence both dosage and toxicant exposure, emphasizing the need for continued research to develop safer cannabis vaping products. Isotopic labeling of myrcene and subsequent trapping via ATD-GC-MS allowed for the elucidation of a novel reaction mechanism that accounted for a significant percentage of the total aerosol VOCs from myrcene. It was also discovered that in addition to the myrcene:THC ratio’s observed effect on the degradation product ratio, the method of vaporization had an effect on the ratio, with vaping yielding lower isoprene degradation products when compared to dabbing.

Between 2019 and early 2020, the EVALI epidemic hospitalized over 2,800 individuals and resulted in 68 deaths, with vitamin E acetate (VEA) strongly linked to the outbreak. During this period, analysis of unknown cannabis concentrate (CC) additives also included pine rosin, medium chain triglycerides (MCT oil), and the hypnotic agent, oleamide. Pine rosin, a known respiratory irritant and occupational asthma trigger, was detected at levels vastly exceeding inhalation safety limits, and exposure to its main component, abietic acid, has been associated with lung injury patterns similar to those observed in EVALI cases.

Ketene, a highly reactive and dangerous respiratory poison, was emitted from vaping THC acetates including Δ^8^-THCO and CBNO, under realistic conditions. The use of isotopically labeled compounds and kinetic trapping experiments confirmed ketene formation at common vaping temperatures (250°C), with oxygen and hydroxyl radicals playing key roles. These findings determined that ketene emissions can form not only from cannabinoid acetates but also from other common e-liquid esters such as ethyl acetate and geranyl acetate, raising broader health concerns about vaping emissions.

As in the case of ENDS vaping, CC vaping is not without risks. Cannabinoids and terpenes are thermally labile. Upon heating and aerosolization, they react to produce toxic emissions. Understanding the origins of toxicant formation is a vital step towards targeted regulatory action and harm mitigation. However, to date, the development of novel cannabis inhalation products has outpaced both basic and applied research. Ongoing vigilance and public awareness based on evidence-based data are needed to help ensure that CC products are free of harmful additives such as pine rosin, THCO, VEA and other injurious compounds.

The results described herein are expected to differ from studies of other formulations such as those that include solvents and other additives. Ongoing issues to address include how chemical formulation and device alterations impact toxic emission production. In addition, an area of need is the investigation of potential synergistic or antagonistic toxicity due to chemical and biological interactions of CC chemical components (Baldovinos et al., 2023; Strongin et al., 2024). Selective chemical sensors amenable to high-throughput screening could aid more effective screening of harmful CC ingredients and emissions. More research must also include user preferences such as vaping topography and related issues to better inform laboratory simulations and toxicological assessments. Chemical studies will continue to remain an enduring need in the CC and related vaping fields to support multidisciplinary and clinical studies with information on dosing (aerosol transfer efficiency), aerosol toxicant identities and levels. Such studies will enable the correlation of variables, such as device characteristic, routes of exposure, etc., to toxicological outcomes.
