# Supplementary material for: Cannabis concentrate vaping chemistry
Source: Front Toxicol. 2025 Jun 9;7:1568207. doi: 10.3389/ftox.2025.1568207 (PMC12183170; doi:10.3389/ftox.2025.1568207)
Supplement: Supplementary file 1 [file DataSheet1.pdf]

Supporting information

Cannabis Concentrate Vaping Chemistry

Kaelas R. Munger, Killian M. Anreise, Robert M Strongin[*]

Department of Chemistry, Portland State University, Portland, Oregon 97217, United States

*** Correspondence:**
Dr. Robert M. Strongin
Strongin@pdx.edu

Table S1

Entry	Comments	Reference
**Molecular composition and chemistry of vaped aerosols from concentrated cannabis (CC) samples**		
**1**	Profiled total VOCs, CO and CO_2 derived from Δ8-THC/VEA aerosols, as well as nicotine vapes. Particle size distributions and the *in vitro* toxicity of the aerosols were studied.	[1]
**2**	Quantified carbonyls, organic acids, cannabinoids, and terpenes in the vaping aerosol of pure VEA, purified THC oil, and an equal volume mixture of VEA and THC oil at various coil temperatures (100-300 degrees C). It was found that degradation of VEA and cannabinoids, including Δ9-THC and cannabigerol (CBG), occurred via radical oxidation and direct thermal decomposition pathways. Evidence of terpene degradation was also observed.	[2]
**3**	Measured CBDQ and other select cannabinoids in nonvaped and vaped commercial CBD pure distillate oil. CBDQ was present in all commercial CBD products and significantly increased after vaping. CBDQ formed adducts with human bronchial epithelial cell proteins including Keap1 and activates KEAP1-Nrf2 stress response pathway genes.	[3]
**4**	Studied the mechanisms of CBD/counterfeit vape cartridge aerosol induced inflammatory response by evaluating the generation of reactive oxygen species by MCT, VEA, and cartridges and their effects on the inflammatory state of pulmonary epithelium and immune cells both *in vitro* and *in vivo.*	[4]
**5**	Targeted profiling of four aerosol carbonyls from EVALI patient samples. Used a commercially available sorbent bed treated with a derivatization solution to trap and stabilize reactive carbonyls in aerosol emissions to reduce reactive analyte losses	[5]

	and improve quantification. Differentiated THC versus nicotine samples. However, a broad spectrum of samples with varying liquid compositions was studied including cannabis-related active ingredients diluted in vitamin E acetate, medium chain triglycerides, squalene, and other nonpolar solvents used to develop an analytical method.	
**6**	Focused on “vape oil liquid” heterogeneous samples and on identifying liquid and aerosol cannabinoids and terpenes. Identified > 100 terpenes and natural extracts, 19 cannabinoids, and other potential toxic additives such as vitamin E acetate, polyethylene glycols, and medium chain triglycerides. Determined more terpenes and minor cannabinoids can be produced via vaporizing and aerosolizing the vape oil. Δ^9-THC and potential toxic additives were found at lower levels in the vapor and aerosol than in the vape liquid.	[6]
**Molecular composition and chemistry of vaped aerosols from dilute cannabis samples**		
**7**	Used diluted samples (≤ 10%) for vaping versus smoking toxicant exposure comparisons.	[7]
**8**	CBD extracts in ethanol diluted with glycerol and vaped. During CBDA vaping most of the acidic CBDA was converted to neutral CBD and further degraded to produce several thermal products. Δ8-iso and Δ9-THC isomers, and cannabichromene (CBC) and CBD quinone (CBDQ) were observed from the vaping process of CBDA and CBD. In the case of Δ9-THC psychoactive hexahydrocannabinol (HHC) derivatives and cannabinol (CBN), were produced via thermal reduction and oxidation.	[8]
**9**	Cannabinoids were diluted to 50 mg/mL in propylene glycol/glycerol solutions. Cannabinoids significantly increased aerosol and carbonyl formation. Most cannabinoids formed hydroxyquinones during vaping. CBD solutions produced significantly higher concentrations of formaldehyde, acetaldehyde, acrolein, diacetyl, and methylglyoxal compared with the other	[9]

	cannabinoid samples. CBG produced higher levels of acetone, methacrolein, and methylglyoxal.	
	**Profiling CC chemical compositions, liquids only, no vaping**	
**10**	Edibles, *C*. sativa flower, concentrates and tinctures were analyzed in their native forms without any sample pretreatment steps prior to analysis by DART-HRMS.	[10]
**11**	GC-MS cannabinoid profiles obtained from vaping liquids including those from EVALI patients.	[11]
**12**	Studied THC levels and potencies in cannabis products.	[12]
**13**	Studied measurement errors in determining cannabis potency	[13]
**14**	Describes validation of a liquid chromatography–diode array detection (LC–DAD) method for quantification of 12 major cannabinoids in Cannabis dried plant materials, concentrates, and oils.	[14]
**15**	Analyses of 38 samples including initial New York cases of EVALI. The illicit fluids had relatively low cannabinoid content, sometimes with unusual Δ9-/Δ8-tetrahydrocannabinol ratios, sometimes containing pesticides and many containing diluents. A notable diluent was VEA, which was found in 64% of the cannabinoid-containing fluids.	[15]
**16**	Of 70 e-liquids, 77.1% of the e-liquids tested underestimated or overestimated the CBD quantities stated in the product labelling.	[16]
**17**	Analyzed pure cannabidiol (CBD), full-spectrum, samples with CBD contents ranging from ca. 24 to 73%, and highly concentrated Δ9- THC samples with contents of ca. 28− 69%. Density, viscosity, fusion enthalpy and melting point of CBD were determined.	[17]
**18**	Twenty Δ8-THC products were purchased from retail stores in Pittsburgh, PA, USA. The range of	[18]

	Δ8-THC content on independent analysis was wide and weakly correlated to the advertised content.	
**19**	Analyzed 284 e-cigarettes from EVALI patients. 82 were nicotine-containing pods, and 202 contained cannabis oil from unauthorized or black-market dealers. Cannabis cartridges had lower levels of THCs and total cannabinoids, and contained significant levels of diluents including (mainly) VEA, medium-chain triglycerides, polyethylene glycol, and castor oil.	19
**20**	Studied counterfeit cartridges and identified vitamin E acetate (VEA) and tetrahydrocannabinol (THC), as well as solvent-derived hydrocarbons, silicon conjugated compounds, various terpenes, pesticides/plasticizers/polycaprolactones, and metals.	20
**21**	Measured the aromatic properties of cannabis flowers and concentrated extracts using comprehensive two-dimensional gas chromatography equipped with time-of-flight mass spectrometry, flame ionization detection, and sulfur chemiluminescence. A new family of volatile sulfur compounds were discovered as significant contributors to the cannabis scent.	21
	Found the cannabimimetic, 5F-ADB, and dextromethorphan in commercially available CBD samples.	22
**22**	A commercial Δ8-THC distillate was found to contain eleven impurities including Δ4,8-iso-THC, Δ4-iso-THC, Δ8-cis-iso-THC, 4,8-epoxy-iso-THC, 8-hydroxy-iso-THC, 9β hydroxyhexahydrocannabinol, 9α-hydroxyhexa-hydrocannabinol, iso-tetrahydrocannabifuran, cannabicitran, olivetol, and Δ9-THC.	23
**23**	A commercial sample of hexahydrocannabiphorol (HHCP) was found to contain impurities not associated with plant material; therefore, showing that HHCP was produced purely synthetically.	24
**24**	Among 43 disposable vape pen devices, cannabinoids such as tetrahydrocannabinol (THC) or cannabidiol (CBD) were identified in 39 of 43	25

	(90.1%) samples, of which three contained both nicotine and THC.	
**25**	The aim of the study was to examine two commercially available electronic cigarette liquid products. Typical cannabinoids and terpenes were identified. An unknown cannabinoid was identified to be Δ9-tetrahydrocannabihexol acetate (Delta(9)-THCH-O. Despite products labeled to contain "60% HHCPM" and "80% 10-OH-HHC," respectively, no such compounds were detected.	[26]
**26**	Series of studies using a variety of analytical techniques to extensive profiles of a variety of cannabinoids and derivatives in commercial products.	[27], [28], [29]
	**Vitamin E acetate and other diluents**	
**27**	Significant ROS generation by VEA vaping emissions was observed in both acellular and cellular systems. Vaped VEA caused oxidative damage and acute lung injury; the effects are more profound than exposure to equivalent concentrations of DQ alone.	[30]
**28**	Investigated the role of the presence of molecular oxygen (O_2) and transition metals in promoting thermal oxidation of e-liquids, resulting in greater degradation than predicted by pure pyrolysis.	[31]
**29**	Investigated the emission product distribution of VEA produced at temperatures ranging from 176 to 356 degrees C. VEA degradation was found to be greatly enhanced with increasing temperature, resulting in a shift towards the production of lower molecular weight compounds, such as the redox active duroquinone (DQ) and short-chain alkenes. Low temperature vaping of VEA resulted in the production of long-chain molecules, such as phytol.	[32]
**30**	Vaporized VEA produced 2,3,5,6-tetramethyl-1,4-benzoquinone and 2,6,10,14-tetramethyl-1-	[33]

	pentadecene. Identification of these degradants will allow future studies to quantify and examine the degradants in vivo and in vitro as biomarkers for exposure and toxicity assessment	
**31**	VEA, vitamin E, coconut, and medium chain triglyceride (MCT) oil were aerosolized. The presence of some identified aerosol chemicals are known respiratory irritants and acute respiratory toxins.	34
**32**	Confirmed the formation of ketene when VEA was vaped. The production of ketene increased with repeat puffs and showed a correlation to temperatures (200 to 500 °C) measured within vaping devices. Heating temperature plays an important role in ketene formation. In addition to ketene, the organic oxidant duroquinone was also obtained as another thermal degradation product of VEA.	35
**33**	The objective of this study was to assess particle size distribution of VEA aerosolized using several types of vaping pens under two puffing regimens and to conduct preliminary chemical analysis of the VEA aerosol to determine if thermal degradation took place during vaping.	36
**34**	A rapid method for the identification of CC additives and diluents: includes EVALI patient samples	37
	**Indoor vaping, particle size distributions**	
**35**	Measured PM2.5 in the retail and consumption space of a cannabis store (a dispensary), where smoking was banned, but vaporizing and dabbing were permitted.	38
**36**	Commercial cartridges containing marijuana liquid of varying CBD:THC ratios were examined. Measured exhaled aerosol within experimental rooms in two inhabited homes, collecting data on peak concentrations, decay and removal rates, and source emissions. These parameters allowed a simple exposure model to be developed. An 8-day experiment with a single puff each day showed elevated concentrations in the small experimental	39

	room for the next 9 h. Mean concentrations during these hours were 63 $\mu g/m^3$, compared to 4.5 $\mu g/m^3$ at other times.	
**37**	Cannabis vape liquids from nine cartridges were analyzed for several metal-bearing particles (Fe, Ni, Al, Co, Cu, Cr, V, Zn, Pb, and Sn). All samples contained a substantial number of NPs, with three samples having a number of particles one to two orders of magnitude higher than the rest of the studied samples, and the particle size distribution was, in most instances, below 150 nm for all metals.	40
**38**	Metal particles in vape liquids were observed by scanning electron microscopy, and laser ablation inductively coupled plasma mass spectrometry confirmed the presence of copper-, zinc-, lead-, and manganese-bearing particles, metals that are in common alloys that may be used to make vape devices. Colocalized particles containing aluminum, silica, and sodium were also detected. These results suggest that metal particles could be a contributing factor to poor measurement precision.	41
	**Storage and degradation chemistry**	
**39**	Examined emissions from cannabis smoking and vaping within an indoor environment by analyzing the gas-phase composition of the indoor air. Vaping primary indoor concentration enhancements were comparable to or higher than smoking values up to a factor of 4 among monoterpenoids. Sesquiterpenoids and some monoterpenoids showed cannabis emissions' thirdhand transport effects as concentration enhancements in the secondary indoor space.	42
**40**	Thermal degradation of CBD, D9-THC, and formation of CBN was studied at $50^{o}C$, $60^{o}C$, $70^{o}C$, and $80^{o}C$ for dried cannabis resin. The effect of pH and temperature on cannabinoid transformations was also examined. The major cannabinoids (CBD, CBN, and Δ9-THC) reacted relatively quickly at high temperature and in an acidic solution. Onset of degradation of CBD, CBN, and Δ9-THC was	43

	achieved by using low temperature, slightly to moderately acidic pH values, and short-time processing.	
**41**	The results of this study indicate that levels of CBD, THC, and CBN within vape liquids consumers purchase can change dramatically depending on the storage conditions (time and temperature) of the vape liquids.	44
	**Device Parameters**	
**42**	Infrared thermography was used to measure heating coil temperatures in one temperature-controlled and two voltage-controlled systems. The cartridges were modified for direct line-of-sight on the heating coils, the wick and coils were saturated with cannabis extract, and fixtures were developed to force two liters per minute air flow past the coils for the full duration of the puff allowed by the device. Voltage alone was a poor indicator of coil temperature and only the temperature-controlled system consistently maintained temperatures less than 400 degrees C for the full puff duration.	45
**43**	Investigated the composition and internal components of 2019 EVALI patient THC and nicotine devices from 2016–19, specifically the metal, ceramic, and polymer components likely to be exposed to heat by disassembling forty-eight components from eight used and unused vaping devices under a microscope and analyzed them using X-ray fluorescence, scanning electron microscopy, and Fourier-transform infrared micro-spectroscopy. The combination of ceramics, metals, and high temperatures in newer THC cartridges is consistent with conditions hypothesized to produce VEA reactions during vaping.	46

(1) Marrocco, A.; Singh, D.; Christiani, D. C.; Demokritou, P. E-Cigarette (E-Cig) Liquid Composition and Operational Voltage Define the In Vitro Toxicity of

Δ8Tetrahydrocannabinol/Vitamin E Acetate (Δ8THC/VEA) E-Cig Aerosols. *Toxicological Sciences* **2022**, *187* (2), 279-297. DOI: 10.1093/toxsci/kfac047 (acccessed 4/23/2025).
(2) Li, Y. C.; Dai, J. Y.; Tran, L. N.; Pinkerton, K. E.; Spindel, E. R.; Nguyen, T. B. Vaping Aerosols from Vitamin E Acetate and Tetrahydrocannabinol Oil: Chemistry and Composition. *Chemical Research in Toxicology* **2022**, *35* (6), 1095-1109. DOI: 10.1021/acs.chemrestox.2c00064.
(3) Love, C. A.; Kim, H. Y. H.; Tallman, K. A.; Clapp, P. W.; Porter, N. A.; Jaspers, I. Vaping Induced Cannabidiol (CBD) Oxidation Product CBD Quinone Forms Protein Adducts with KEAP1 and Activates KEAP1-Nrf2 Genes. *Chemical Research in Toxicology* **2023**, *36* (4), 565-569. DOI: 10.1021/acs.chemrestox.3c00038.
(4) Muthumalage, T.; Lucas, J. H.; Wang, Q. X.; Lamb, T.; McGraw, M. D.; Rahman, I. Pulmonary Toxicity and Inflammatory Response of Vape Cartridges Containing Medium-Chain Triglycerides Oil and Vitamin E Acetate: Implications in the Pathogenesis of EVALI. *Toxics* **2020**, *8* (3). DOI: 10.3390/toxics8030046.
(5) McGuigan, M.; Chapman, G.; Lewis, E.; Watson, C. H.; Blount, B. C.; Valentin-Blasini, L. High-Performance Liquid Chromatography–Tandem Mass Spectrometry Analysis of Carbonyl Emissions from E-Cigarette, or Vaping, Products. *ACS omega* **2022**, *7* (9), 7655-7661.
(6) Guo, W. H.; Vrdoljak, G.; Liao, V. C.; Moezzi, B. Major Constituents of Cannabis Vape Oil Liquid, Vapor and Aerosol in California Vape Oil Cartridge Samples. *Frontiers in Chemistry* **2021**, *9*. DOI: 10.3389/fchem.2021.694905.
(7) Sambiagio, N.; Iria, D. A. G.; Auer, R.; Schöni, A.; Berthet, A. Toxicological assessment of aerosols emitted by cannabis inhalation methods: Does cannabis vaping using Electronic Non-Nicotine Delivery Systems (ENNDS) and vaporizers reduce exposure to toxicants compared to cannabis smoking? *Federal Office of Public Health* **2023**.
(8) Kim, E. J.; Kwon, E.; Oh, S. J.; Choi, M. R.; Lee, S. R.; Jung, B. H.; Lee, W.; Hong, J. Thermal transformation of CBD, CBDA, and Δ9-THC during e-cigarette vaping: Identification of conversion products by GC-MS. *Journal of Chromatography A* **2025**, *1749*. DOI: 10.1016/j.chroma.2025.465909.
(9) Robertson, N. E.; Connolly, J.; Shevchenko, N.; Mascal, M.; Pinkerton, K. E.; Nicklisch, S. C. T.; Nguyen, T. B. Chemical Composition of Aerosols from the E-Cigarette Vaping of Natural and Synthetic Cannabinoids. *Chemical Research in Toxicology* **2024**, *37* (12), 1965-1975. DOI: 10.1021/acs.chemrestox.4c00326.
(10) Chambers, M. I.; Musah, R. A. DART-HRMS triage approach part 2–application to the detection of cannabinoids and terpenes in recreational Cannabis products. *Forensic chemistry* **2023**, *33*, 100469.
(11) Ciolino, L. A.; Ranieri, T. L.; Brueggemeyer, J. L.; Taylor, A. M.; Mohrhaus, A. S. EVALI vaping liquids part 1: GC-MS cannabinoids profiles and identification of unnatural THC isomers. *Frontiers in Chemistry* **2021**, *9*, 746479.
(12) Glinn, M. A.; Michaud, G. P. Potency levels of regulated cannabis products in Michigan 2021–2022. *Journal of forensic sciences* **2023**, *68* (6), 1894-1905.
(13) Goldman, S. Measurement uncertainty revealed: The impacts of Certified Reference Material (CRM) on cannabinoid concentrations in the cannabis testing industry. *Talanta Open* **2024**, *10*, 100344.
(14) Vaclavik, L.; Benes, F.; Fenclova, M.; Hricko, J.; Krmela, A.; Svobodova, V.; Hajslova, J.; Mastovska, K. Quantitation of Cannabinoids in Cannabis Dried Plant Materials, Concentrates,

and Oils Using Liquid Chromatography–Diode Array Detection Technique with Optional Mass Spectrometric Detection: Single-Laboratory Validation Study, First Action 2018.11. *Journal of AOAC INTERNATIONAL* **2019**, *102* (6), 1822-1833. DOI: 10.1093/jaoac/102.6.1822 (acccessed 4/14/2025).
(15) Duffy, B.; Li, L. Y.; Lu, S. J.; Durocher, L.; Dittmar, M.; Delaney-Baldwin, E.; Panawennage, D.; LeMaster, D.; Navarette, K.; Spink, D. Analysis of Cannabinoid-Containing Fluids in Illicit Vaping Cartridges Recovered from Pulmonary Injury Patients: Identification of Vitamin E Acetate as a Major Diluent. *Toxics* **2020**, *8* (1). DOI: 10.3390/toxics8010008.
(16) Dunn, K.; Taylor, A.; Turfus, S. A review of cannabidiol-containing electronic liquids-Current regulations and labelling accuracy. *Drug Testing and Analysis* **2021**, *13* (8), 1490-1498. DOI: 10.1002/dta.3102.
(17) Idárraga-Vélez, A. M.; Chaves, I. D. G.; Orozco, G. A. Densities and Viscosities of Cannabis Extracts and Distillates, and Densities, Viscosities, Fusion Enthalpy, and Melting Point of Cannabidiol. *Journal of Chemical and Engineering Data* **2023**, *68* (12), 2982-2988. DOI: 10.1021/acs.jced.3c00105.
(18) Kaczor, E. E.; Greene, K.; Babu, K. M.; Berthold, E. C.; Sharma, A.; Carreiro, S. P. Commercial delta-8 THC products: an analysis of content and labeling. *Journal of Medical Toxicology* **2024**, *20* (1), 31-38.
(19) Lu, S.; Li, L.; Duffy, B. C.; Dittmar, M. A.; Durocher, L. A.; Panawennage, D.; Delaney-Baldwin, E. R.; Spink, D. C. Investigation of vaping fluids recovered from New York State e-cigarette or vaping product use-associated lung injury patients. *Frontiers in chemistry* **2021**, *9*, 748935.
(20) Muthumalage, T.; Friedman, M. R.; McGraw, M. D.; Ginsberg, G.; Friedman, A. E.; Rahman, I. Chemical constituents involved in e-cigarette, or vaping product use-associated lung injury (EVALI). *Toxics* **2020**, *8* (2), 25.
(21) Oswald, I. W. H.; Ojeda, M. A.; Pobanz, R. J.; Koby, K. A.; Buchanan, A. J.; Del Rosso, J.; Guzman, M. A.; Martin, T. J. Identification of a New Family of Prenylated Volatile Sulfur Compounds in <i>Cannabis</i> Revealed by Comprehensive Two-Dimensional Gas Chromatography. *Acs Omega* **2021**, *6* (47), 31667-31676. DOI: 10.1021/acsomega.1c04196.
(22) Poklis, J. L.; Mulder, H. A.; Peace, M. R. The unexpected identification of the cannabimimetic, 5F-ADB, and dextromethorphan in commercially available cannabidiol e-liquids. *Forensic science international* **2019**, *294*, e25-e27.
(23) Radwan, M. M.; Wanas, A. S.; Gul, W.; Ibrahim, E. A.; ElSohly, M. A. Isolation and Characterization of Impurities in Commercially Marketed Δ8-THC Products. *Journal of Natural Products* **2023**, *86* (4), 822-829. DOI: 10.1021/acs.jnatprod.2c01008.
(24) Schirmer, W.; Gjuroski, I.; Vermathen, M.; Furrer, J.; Schürch, S.; Weinmann, W. Isolation and characterization of synthesis intermediates and side products in hexahydrocannabiphorol. *Drug Testing and Analysis* **2024**. DOI: 10.1002/dta.3759.
(25) Wang, P.; Williams, R. J.; Chen, W.; Wang, F.; Shamout, M.; Tanz, L. J.; Herzig, C. T.; Oakley, L. P.; Peak, C. M.; Heinzerling, A. Chemical composition of electronic vaping products from school grounds in California. *Nicotine and Tobacco Research* **2024**, *26* (8), 991-998.
(26) Watanabe, S.; Murakami, T.; Muratsu, S.; Fujiwara, H.; Nakanishi, T.; Seto, Y. Discrepancies between the stated contents and analytical findings for electronic cigarette liquid products: Identification of the new cannabinoid, Δ9-tetrahydrocannabihexol acetate. *Drug Testing and Analysis* **2024**. DOI: 10.1002/dta.3777.

(27) Tanaka, R.; Ito, M.; Kikura-Hanajiri, R. Identification of Acetyl, Propionyl, and Butanoyl Derivatives of THC and Its Analogs, and Their Synthetic By-Products in Oils and a Herbal Product. *Drug Testing and Analysis* **2025**. DOI: 10.1002/dta.3883.
(28) Tanaka, R.; Kikura-Hanajiri, R. Identification of hexahydrocannabinol (HHC), dihydro-iso-tetrahydrocannabinol (dihydro-iso-THC) and hexahydrocannabiphorol (HHCP) in electronic cigarette cartridge products. *Forensic Toxicology* **2024**, *42* (1), 71-81. DOI: 10.1007/s11419-023-00667-9.
(29) Tanaka, R.; Kikura-Hanajiri, R. Identification of Δ8-Tetrahydrocannabinol (THC) and Δ9-THC Analogs, with Different Lengths of Alkyl Chain at C-3 Position, in Oil Products Distributed on the Internet. *Yakugaku Zasshi-Journal of the Pharmaceutical Society of Japan* **2024**, *144* (8), 823-837.
(30) Canchola, A.; Ahmed, C. M. S.; Chen, K. P.; Chen, J. Y.; Lin, Y. H. Formation of Redox-Active Duroquinone from Vaping of Vitamin E Acetate Contributes to Oxidative Lung Injury. *Chemical Research in Toxicology* **2022**, *35* (2), 254-264. DOI: 10.1021/acs.chemrestox.1c00309.
(31) Canchola, A.; Langmo, S.; Meletz, R.; Lum, M.; Lin, Y. H. External Factors Modulating Vaping-Induced Thermal Degradation of Vitamin E Acetate. *Chemical Research in Toxicology* **2023**, *36* (1), 83-93. DOI: 10.1021/acs.chemrestox.2c00298.
(32) Canchola, A.; Meletz, R.; Khandakar, R. A.; Woods, M.; Lin, Y. H. Temperature dependence of emission product distribution from vaping of vitamin E acetate. *Plos One* **2022**, *17* (3). DOI: 10.1371/journal.pone.0265365.
(33) Kovach, A. L.; Carter, R. R.; Thornburg, J. W.; Wiethe, R.; Fennell, T. R.; Wiley, J. L. Thermal Degradants Identified from the Vaping of Vitamin E Acetate. *Journal of Analytical Toxicology* **2022**, *46* (7), 750-756. DOI: 10.1093/jat/bkab109.
(34) LeBouf, R. F.; Ranpara, A.; Ham, J.; Aldridge, M.; Fernandez, E.; Williams, K.; Burns, D. A.; Stefaniak, A. B. Chemical Emissions From Heated Vitamin E Acetate-Insights to Respiratory Risks From Electronic Cigarette Liquid Oil Diluents Used in the Aerosolization of Δ9-THC-Containing Products. *Frontiers in Public Health* **2022**, *9*. DOI: 10.3389/fpubh.2021.765168.
(35) Wang, P.; Jacob, P.; Wang, Z. M.; Fowles, J.; O'Shea, D. F.; Wagner, J.; Kumagai, K. Conditions Leading to Ketene Formation in Vaping Devices and Implications for Public Health. *Chemical Research in Toxicology* **2024**, *37* (8), 1415-1427. DOI: 10.1021/acs.chemrestox.4c00190.
(36) Mikheev, V. B.; Klupinski, T. P.; Ivanov, A.; Lucas, E. A.; Strozier, E. D.; Fix, C. Particle size distribution and chemical composition of aerosolized vitamin E acetate. *Aerosol Science and Technology* **2020**, *54* (9), 993-998. DOI: 10.1080/02786826.2020.1783431.
(37) Jiang, H. H.; Ahmed, C. M. S.; Martin, T. J.; Canchola, A.; Oswald, I. W. H.; Garcia, J. A.; Chen, J. Y.; Koby, K. A.; Buchanan, A. J.; Zhao, Z. X.; et al. Chemical and Toxicological Characterization of Vaping Emission Products from Commonly Used Vape Juice Diluents. *Chemical Research in Toxicology* **2020**, *33* (8), 2157-2163. DOI: 10.1021/acs.chemrestox.0c00174.
(38) Murphy, M. B.; Huang, A. S.; Schick, S. F. PM 2.5 concentrations in a cannabis store with on-site consumption. *Environmental health perspectives* **2021**, *129* (6), 067701.
(39) Wallace, L.; Ott, W.; Zhao, T. Secondhand exposure from vaping marijuana: Concentrations, emissions, and exposures. **2020**.
(40) Gajdosechova, Z.; Marleau-Gillette, J. Single particle analysis of polydisperse metal-bearing particles in cannabis vape liquids by organic mode ICP-MS. *Journal of Analytical Atomic Spectrometry* **2024**, *39* (6), 1482-1492. DOI: 10.1039/d4ja00045e.

(41) Gajdosechova, Z.; Marleau-Gillette, J.; Turnbull, M. J.; Petts, D. C.; Jackson, S. E.; Cabecinha, A.; Abramovici, H.; Waye, A.; Melanson, J. E. Evidence That Metal Particles in Cannabis Vape Liquids Limit Measurement Reproducibility. *Acs Omega* **2022**, *7* (47), 42783-42792. DOI: 10.1021/acsomega.2c03797.
(42) Askari, A.; Wang, X.; Ortiz, R.; Di Ciano, P.; Hassan, A. N.; Rueda, S.; Chow, C.-W.; Chan, A. W. Measurement of volatile organic compounds from indoor cannabis smoking and vaping: Direct effects and secondary transport. *Indoor Environments* **2024**, *1* (1), 100006.
(43) Jaidee, W.; Siridechakorn, I.; Nessopa, S.; Wisuitiprot, V.; Chaiwangrach, N.; Ingkaninan, K.; Waranuch, N. Kinetics of CBD, Δ9-THC Degradation and Cannabinol Formation in Cannabis Resin at Various Temperature and pH Conditions. *Cannabis and Cannabinoid Research* **2022**, *7* (4), 537-547. DOI: 10.1089/can.2021.0004.
(44) Moreno, S.; Trouten-Ebert, A.; Richards-Waugh, L. L.; Quiñones, R. An evaluation of the cannabinoid content of the liquid and thermal degradation analysis of cannabis-labeled vape liquids. *Journal of Forensic Sciences* **2024**, *69* (3), 905-918. DOI: 10.1111/1556-4029.15508.
(45) Oar, M. A.; Savage, C. H.; Rufer, E. S.; Rucker, R. P.; Guzman, J. A. Thermography of cannabis extract vaporization cartridge heating coils in temperature- and voltage-controlled systems during a simulated human puff. *Plos One* **2022**, *17* (1). DOI: 10.1371/journal.pone.0262265.
(46) Wagner, J.; Chen, W. H.; Vrdoljak, G. Vaping cartridge heating element compositions and evidence of high temperatures. *Plos One* **2020**, *15* (10). DOI: 10.1371/journal.pone.0240613.
